# Glycolytic reprogramming mediated by the ADAM12/IGF1 axis promotes ossification of the posterior longitudinal ligament

**DOI:** 10.1038/s41420-026-03044-8

**Published:** 2026-03-25

**Authors:** Qi Zhao, Hao Wu, Dong Xie, Mingliang Shi, Baocheng Niu, Lili Yang

**Affiliations:** 1 Spine Center, Department of Orthopaedics, Shanghai Changzheng Hospital, Second Affiliated Hospital of Naval Medical University, Shanghai, China; 2 Department of Orthopaedics, Navy 905th Hospital, Naval Medical University, Shanghai, China

**Keywords:** Growth factor signalling, Metabolic disorders

## Abstract

Ossification of the posterior longitudinal ligament (OPLL) is a debilitating spinal disorder characterized by heterotopic bone formation and severe neurological deficits, yet its underlying pathogenic mechanisms remain poorly understood. Here, we report that glycolytic reprogramming orchestrated by the ADAM12/IGF1 axis is a critical driver of OPLL pathogenesis. Through integrated multi-omics analysis of human OPLL tissues, we uncovered a profound metabolic shift featuring enhanced glycolysis and suppressed oxidative phosphorylation as ligament progenitor cells differentiated into osteoblasts. This glycolytic reprogramming was functionally indispensable for ossification, as its inhibition with 2-deoxy-D-glucose robustly attenuated osteogenic differentiation in vitro and ectopic bone formation in vivo. We identified ADAM12 as a pivotal upstream regulator that is highly upregulated in OPLL. Mechanistically, ADAM12 activates the IGF1 signaling pathway by modulating IGFBP5, which in turn drives glycolytic flux and lactate production to fuel the osteogenic differentiation of ligament cells. Crucially, the pro-ossific effects of ADAM12 were reversed by inhibition of either IGF1R or glycolysis. Taken together, our study establishes the ADAM12-IGF1-glycolysis axis as a central pathogenic mechanism and a promising therapeutic target for OPLL.

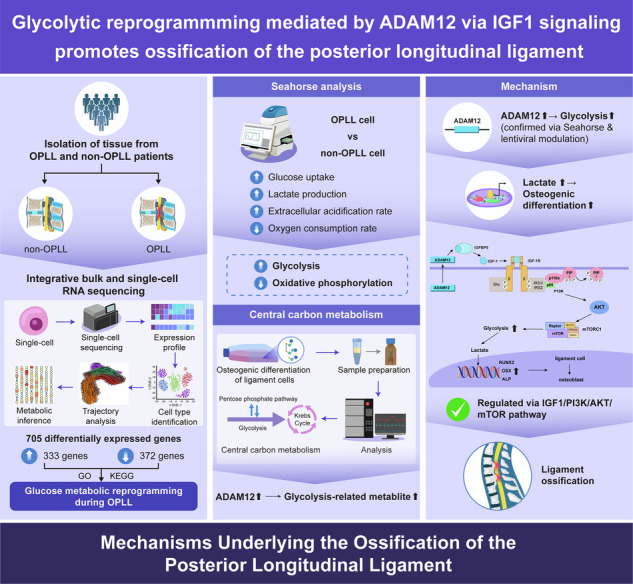

## Introduction

Ossification of the posterior longitudinal ligament (OPLL) is a debilitating spinal disorder characterized by the heterotopic bone formation within the spinal ligament, leading to progressive spinal canal stenosis and severe neurological deficits such as quadriplegia and incontinence [1]. It predominantly affects East Asian populations, with a reported prevalence of up to 18.22% in patients with degenerative cervical spine conditions [2, 3]. While surgical intervention remains the primary treatment for advanced cases, it carries significant risks and fails to halt the progression of ossification, underscoring the urgent need for effective pharmacotherapeutic strategies targeting the underlying pathogenesis [4].

Despite its clinical significance, the pathogenic mechanisms driving OPLL remain poorly understood, hindering the development of targeted therapies. While genetic predisposition [5, 6], non-coding RNAs [7–9], persistent mechanical stress [10, 11], and metabolic comorbidities like diabetes [2, 12] have been implicated, these factors alone offer only a partial explanation. A fundamental, unifying mechanism that serves as a central hub to initiate and sustain the aberrant osteogenic program within ligament tissue is still missing. We postulated that a profound rewiring of cellular metabolism could serve as such a central hub, reprogramming ligament progenitor cells toward a pathological bone-forming phenotype.

This hypothesis is grounded in the critical role of metabolic reprogramming in cell fate determination [13, 14]. In physiological bone formation, differentiating osteoblasts undergo a distinct metabolic switch from oxidative phosphorylation to aerobic glycolysis - a Warburg-like effect [15]. This shift is not merely an alternative energy source but also actively driven by anabolic signals like PTH and Wnt and serves essential purposes [16, 17]. It rapidly generates ATP and, crucially, provides abundant biosynthetic precursors for the massive production of collagen and other bone matrix components [18–20]. Perhaps even more intriguingly, the glycolytic end-product lactate is no longer viewed as mere waste. It has emerged as a signaling metabolite that can promote osteogenic differentiation through epigenetic mechanisms, including histone lactylation [14, 21]. However, despite this well-established paradigm in physiological osteogenesis, whether a pathological metabolic reprogramming governs the ectopic ossification in OPLL has been entirely unexplored. This represents a significant gap in our understanding of OPLL.

To bridge this gap, we conducted an unbiased integrated multi-omics analysis of human OPLL tissues. Our transcriptomic and single-cell RNA sequencing analyses first confirmed a pronounced enrichment of glycolytic pathways specifically within the osteogenic ligament cell lineage. From this metabolic signature, we pinpointed A Disintegrin And Metalloproteinase 12 (ADAM12), specifically its secreted isoform, as a top candidate regulator. ADAM12 was among the most significantly upregulated genes in OPLL, and its expression showed a striking positive correlation with glycolytic activity at both the tissue and single-cell levels. This finding converged with several key strands of evidence. ADAM12 is intimately involved in skeletal development and bone metabolism, with its deletion leading to ossification defects in model organisms [22]. Furthermore, it can modulate the bioavailability of growth factors like IGF1 by cleaving insulin-like growth factor-binding proteins [23, 24]. Notably, ADAM12 has been shown to directly regulate the expression of glycolytic enzymes in other disease contexts, such as cancer [25]. These converging lines of evidence led us to hypothesize that ADAM12 functions as a critical upstream regulator in OPLL, activating relevant signaling pathways to drive a glycolytic metabolic reprogramming, which in turn fuels the pathogenic osteogenic differentiation of ligament cells. In this study, we rigorously test this hypothesis through multi-omics, biochemical, and in vivo approaches, revealing a novel and targetable pathogenic axis in OPLL.

## Results

### Multi-omics profiling uncovers glycolytic reprogramming during ligament ossification

**Fig. 1:**
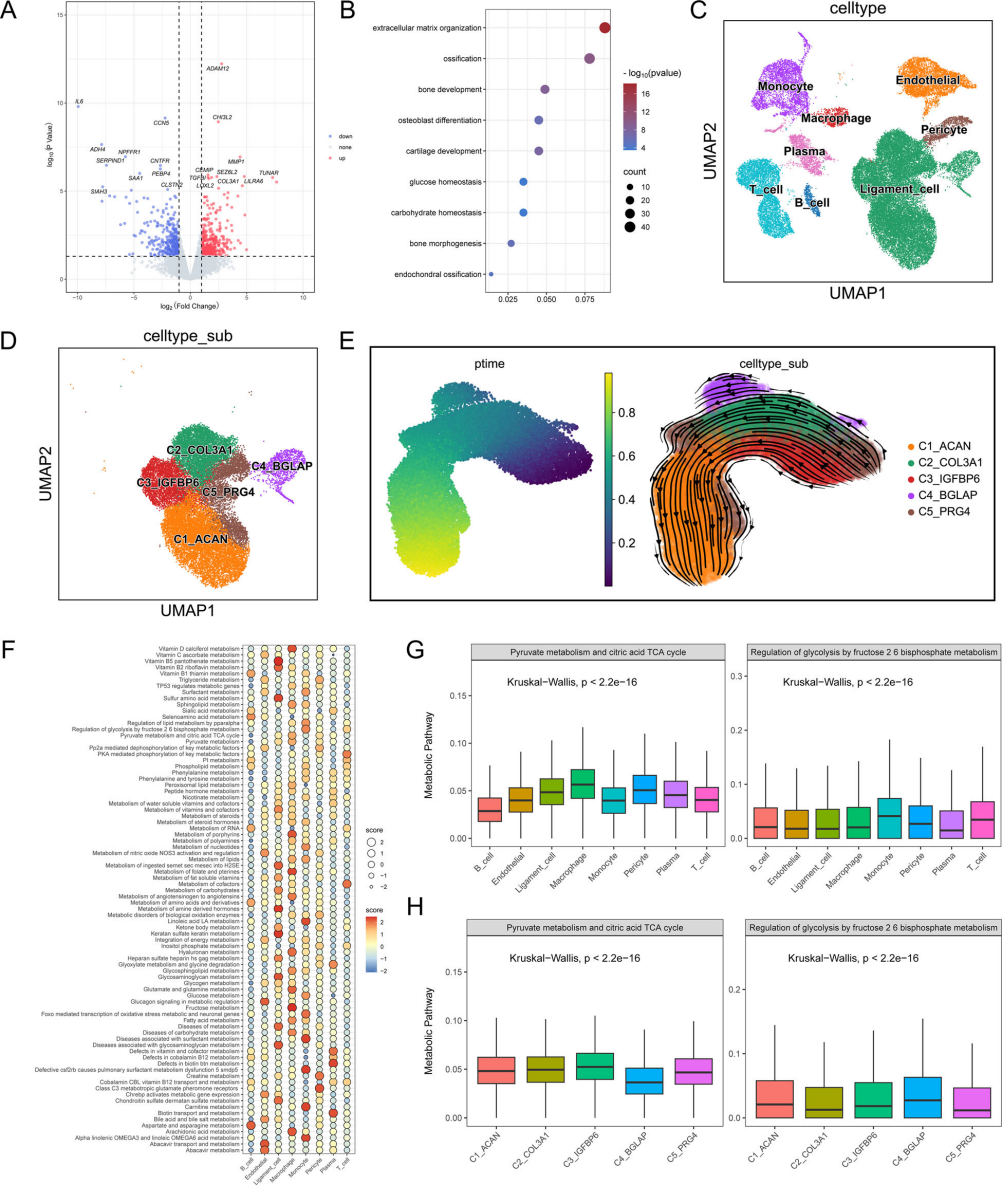
Integrated transcriptomic analyses reveal glycolytic reprogramming during ligament cell differentiation in OPLL. **A** Volcano plots show DEGs between OPLL and non-OPLL samples. Significantly upregulated (red) and downregulated (blue) genes are highlighted. **B** GO enrichment analysis of the DEGs, highlighting significant terms related to glucose metabolism. **C** UMAP visualization of 45,808 single cells from OPLL and non-OPLL tissues, color-coded by annotated cell types. **D** UMAP plot of the ligament cell cluster revealing five distinct subpopulations. **E** Pseudotime trajectory analysis of ligament cells depicting the differentiation path from progenitor (C5) to ossified (C4) and chondrocyte-like (C1) states. **F** Metabolic pathway activity scores across all cell types calculated using ScMetabolism and REACTOME gene sets. **G** Box plots showing the distribution of enrichment scores for representative glycolytic (left) and oxidative phosphorylation (right) pathways across the major cell types. **H** Metabolic dynamics along the ligament cell differentiation trajectory. Enrichment scores of glycolytic (left) and oxidative phosphorylation (right) pathways are projected onto the pseudotime trajectory, illustrating a metabolic switch towards glycolysis during osteogenic differentiation. Data are presented as mean ± standard deviation.

To gain unbiased insights into the pathogenesis of OPLL, we commenced with transcriptomic profiling of human OPLL and non-ossified ligament tissues. Bulk RNA-seq analysis identified 705 differentially expressed genes (DEGs), comprising 333 upregulated genes and 372 downregulated genes (Fig. 1A). These DEGs were subjected to GO/KEGG enrichment analysis. Strikingly, pathway enrichment analysis revealed a significant overrepresentation of terms related to glucose and carbohydrate homeostasis (Fig. 1B, Fig. S1A).

To deconvolute this metabolic signature at cellular resolution, we performed single-cell RNA sequencing analysis on the same tissue types. Unsupervised clustering of 45,808 high-quality cells defined the cellular landscape of the posterior longitudinal ligament, identifying eight major cell types, including ligament cells, endothelial cells, pericytes, monocytes, macrophages, T cells, B cells, and plasma cells (Fig. 1C). Expression profiles of marker genes for each cell type were quantified and matched to known biological annotations (Fig. S1C). Focusing on the ligament cell lineage, we resolved five distinct subpopulations: progenitor cells (C5, PRG4^hi^), chondrocyte-like cells (C1, ACAN^hi^), fibroblasts (C2, COL3A1^hi^ and C3, IGFBP6^hi^), and ossified cells (C4, BGLAP^hi^) (Fig. 1D, Fig. S1B). Pseudotime trajectory inference reconstructed a clear differentiation path from C5 progenitors towards C4 osteoblasts and C1 chondrocytes (Fig. 1E).

We then interrogated the metabolic state of these subpopulations. Single-cell metabolic pathway analysis revealed a compartmentalized distribution of metabolic activities across the ligament (Fig. 1F). Notably, ligament cells were highly enriched for glucose metabolism pathways. Tracking these pathways along the differentiation trajectory unveiled a definitive metabolic switch: as ligament progenitors differentiated into osteoblasts, they exhibited a marked increase in glycolytic process enrichment and a concomitant decrease in oxidative phosphorylation activity (Fig. 1G, H).

### OPLL cells exhibit a glycolytic dependency and compromised mitochondrial respiration

**Fig. 2:**
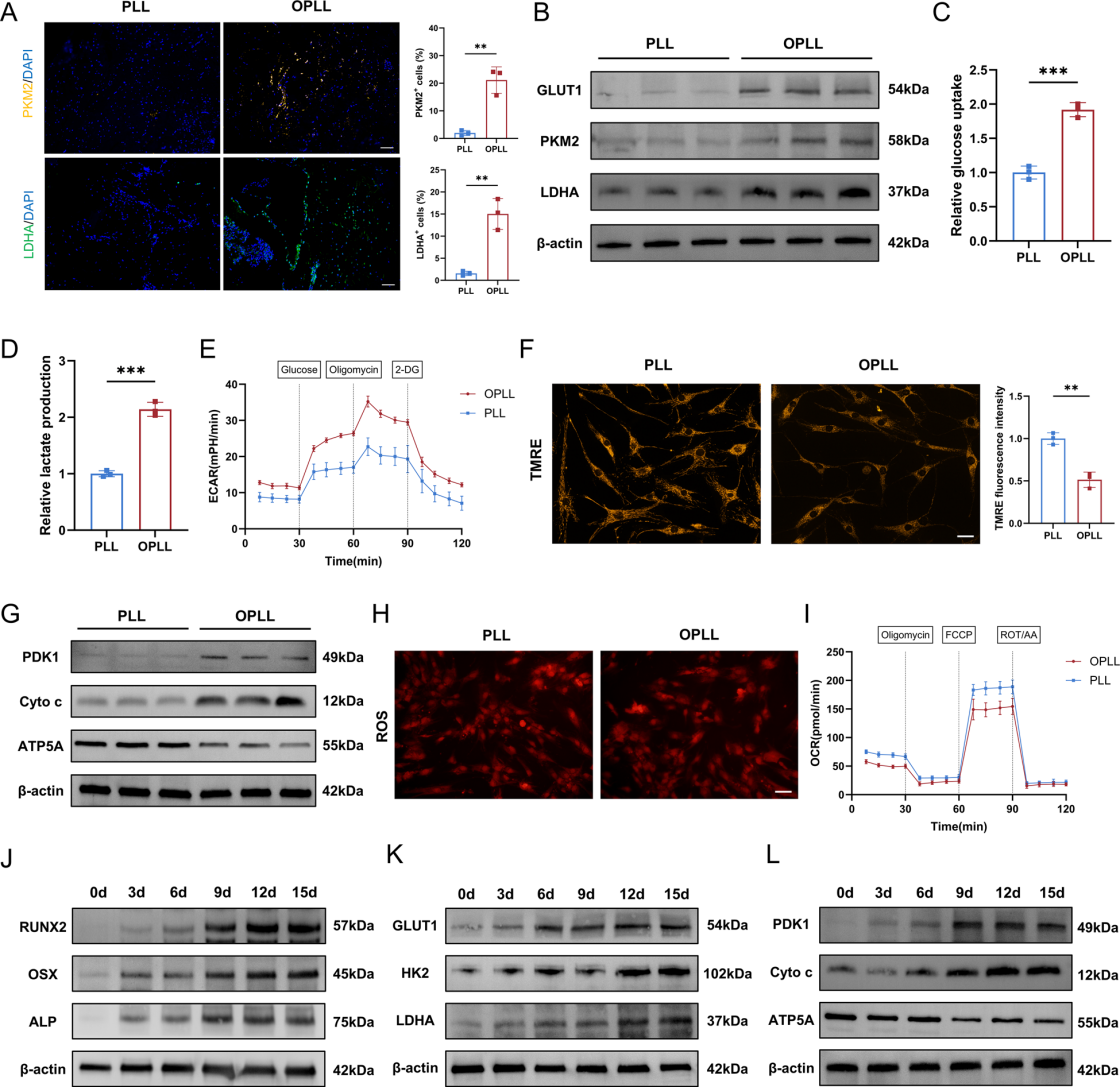
Functional characterization of enhanced glycolysis and impaired oxidative phosphorylation in OPLL. **A** Representative immunofluorescence images and quantification of glycolytic enzymes PKM2 and LDHA in human PLL and OPLL tissues. Scale bar = 100 µm, *n* = 3. **B** Western blot analysis of GLUT1, PKM2, and LDHA protein levels in PLL and OPLL cells. *n* = 3. **C**, **D**. Glucose uptake and lactate production in PLL and OPLL cells. *n* = 3. **E** ECAR measured by Seahorse XF Analyzer to assess the glycolytic capacity of PLL and OPLL cells. *n* = 3. **F** Representative images showing TMRE staining (orange-red) in PLL and OPLL cells. The bar graph depicts the quantification of relative TMRE fluorescence intensity. Scale bar = 30 µm, *n* = 3. **G** Western blot analysis of PDK1, Cyto c, and ATP5A in OPLL and PLL cells. **H** Intracellular ROS levels measured by DCFH-DA fluorescence. Scale bar = 50 µm. **I** OCR measured by Seahorse XF Analyzer to assess mitochondrial respiration. *n* = 3. **J** Western blot analysis of RUNX2, OSX, and ALP during a 15day time course of osteogenic differentiation of ligament cells. **K** Western blot analysis of GLUT1, HK2, and LDHA during a 15day time course of osteogenic differentiation of ligament cells. **L** Western blot analysis of PDK1, Cyto c, and ATP5A during a 15day time course of osteogenic differentiation of ligament cells. Data are presented as mean ± standard deviation.

To functionally characterize the metabolic state of OPLL cells, immunofluorescence staining of human ligament tissues revealed a substantial upregulation of the key glycolytic enzymes PKM2 and LDHA in OPLL lesions compared to non-ossified controls (Fig. 2A). In addition, in OPLL tissues, there was an increase in RUNX2^+^ cells and CD31^+^ vascular regions (Fig. S1D). To quantitatively assess this metabolic shift, ligament cells were isolated from OPLL and normal PLL tissues using the tissue explant technique and identified as PRG4^+^ ligament cells (Fig. S1E, F). OPLL cells exhibited stronger osteogenic ability (Fig. S1G). OPLL cells exhibited markedly elevated protein levels of glucose transporter GLUT1 and glycolytic enzymes PKM2 and LDHA (Fig. 2B, Fig. S2A). Functionally, these cells demonstrated enhanced glucose uptake (Fig. 2C), increased lactate production (Fig. 2D), and a higher extracellular acidification rate (ECAR), a direct measure of glycolytic flux (Fig. 2E). Concurrently, we investigated mitochondrial oxidative metabolism. TMRE staining revealed a significant loss of mitochondrial membrane potential in OPLL cells (Fig. 2F), which was associated with the upregulation of PDK1, increased cytochrome c release, and decreased ATP5A expression (Fig. 2G, Fig. S2B). Notably, this was not accompanied by a change in overall reactive oxygen species (ROS) levels (Fig. 2H). Crucially, Seahorse analysis confirmed a profound reduction in the oxygen consumption rate (OCR) in OPLL cells (Fig. 2I).

To determine if this metabolic state is acquired during the osteogenic process, we tracked metabolic markers during the osteogenic differentiation of ligament cells. While osteogenic markers (RUNX2, OSX, ALP) increased over time (Fig. 2J, Fig. S2C), key glycolytic actors (GLUT1, HK2, LDHA) were progressively upregulated (Fig. 2K, Fig. S2D). Conversely, markers of oxidative phosphorylation (ATP5A) declined, the inhibitory factor PDK1 rose, and cytochrome c release increased (Fig. 2L, Fig. S2E).

### Pharmacological inhibition of glycolysis attenuates ossification in vitro and in vivo

**Fig. 3:**
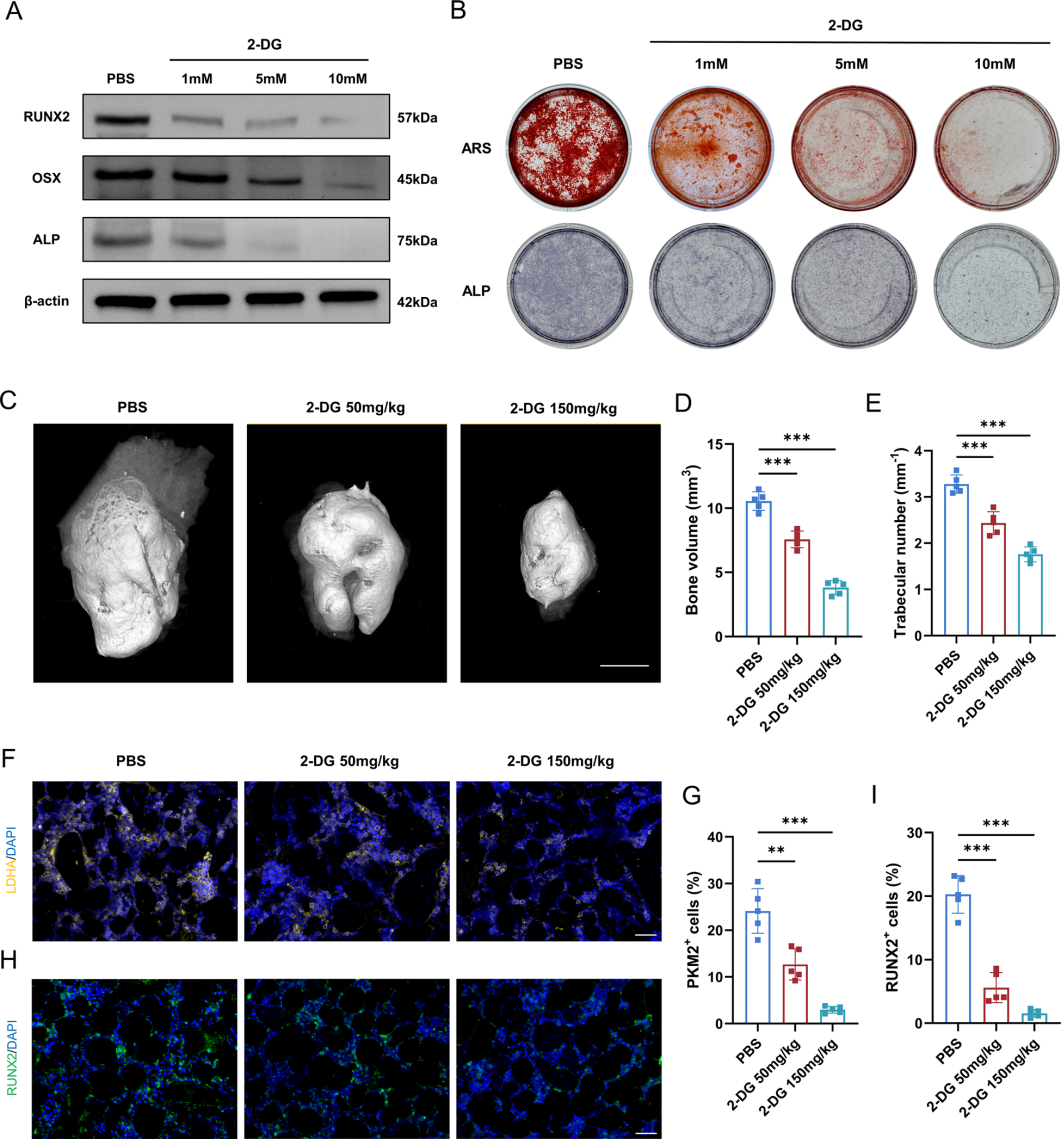
Glycolytic inhibition by 2-DG suppresses ligament cell osteogenesis and ectopic ossification. **A** Western blot analysis of osteogenic markers (RUNX2, OSX, ALP) in ligament cells treated with increasing doses of 2-DG during osteogenic induction. **B** Representative images of ARS and ALP staining of ligament cells under osteogenic induction with the indicated concentrations of 2-DG. **C** Representative three-dimensional micro-CT reconstructions of heterotopic ossification masses from mice implanted with BMP2 and treated with PBS or 2-DG (50 or 150 mg/kg) for 14 days. Scale bar = 2 mm, *n* = 5. **D**, **E** Quantitative analysis of bone volume and trabecular number of the heterotopic ossification sites. *n* = 5. **F**, **G** Representative immunofluorescence images and quantification of PKM2⁺ cells within the heterotopic ossification sites. Scale bar = 50 μm, *n* = 5. **H**, **I** Representative immunofluorescence images and quantification of RUNX2⁺ cells within the heterotopic ossification sites. Scale bar = 50 μm, *n* = 5. Data are presented as mean ± standard deviation.

To test the functional requirement of glycolysis in OPLL pathogenesis, we examined the effects of its inhibition on ossification. Treating ligament cells with the glycolytic inhibitor 2-deoxy-D-glucose (2-DG) during osteogenic induction resulted in a dose-dependent reduction in the expression of key osteogenic markers, RUNX2, OCN, and ALP (Fig. 3A, Fig. S6B). This was accompanied by a marked decrease in matrix mineralization and alkaline phosphatase activity, as evidenced by ARS and ALP staining, respectively (Fig. 3B).

We next translated these findings in vivo using a mouse model of BMP2-induced heterotopic ossification. Systemic administration of 2-DG significantly reduced the size and density of the resulting osteogenic mass in a dose-dependent manner (Fig. 3C). Micro-CT quantification revealed that glycolysis inhibition led to substantial decreases in both bone volume and trabecular number (Fig. 3D, E). Immunofluorescence analysis of the explanted tissues showed that 2-DG treatment significantly reduced the abundance of PKM2⁺ cells (Fig. 3F, G) and RUNX2⁺ osteoprogenitor cells (Fig. 3H, I) within the ossification site.

### ADAM12 is identified as a key metabolic regulator positively correlated with glycolysis in OPLL

**Fig. 4:**
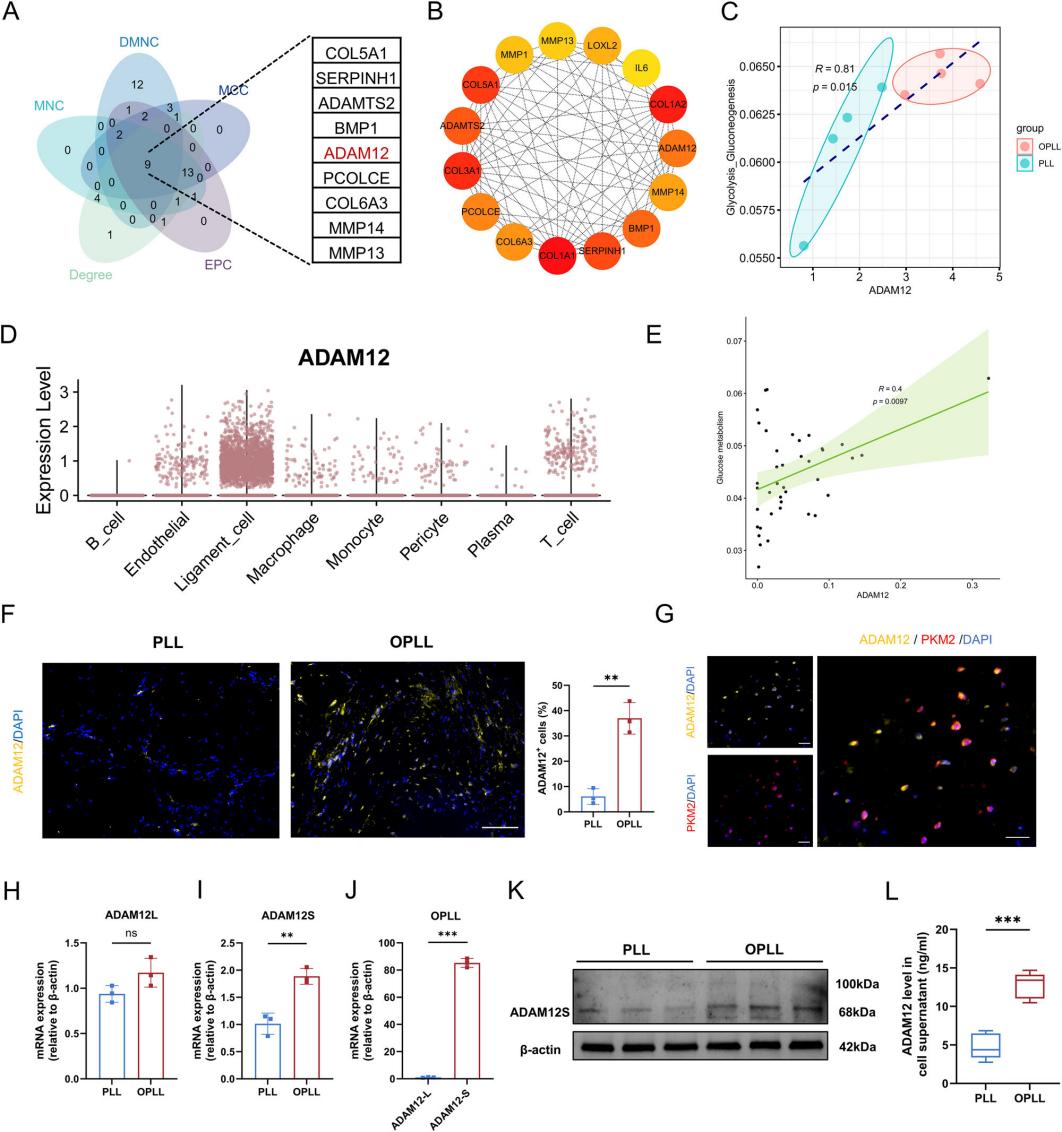
ADAM12 is identified as a key regulator of OPLL, positively correlated with glycolysis. **A** Venn diagram illustrating the overlap of hub genes identified from the PPI network using five different centrality algorithms: MCC, DMNC, EPC, MNC, and Degree. **B** Key genes identified by the DMNC method within the PPI network. **C** Correlation analysis between the glycolysis flux score and ADAM12 expression in OPLL and PLL tissues. **D** Distribution of ADAM12 across the major cell types in posterior longitudinal ligament tissue. **E** Scatter plot showing the positive correlation between ADAM12 expression and the glucose metabolism pathway activity score across individual ligament cells. **F** Representative immunofluorescence images and quantification of ADAM12 expression in human OPLL and PLL tissues. Scale bar = 50 μm, *n* = 3. **G** Representative immunofluorescence images showing co-localization of ADAM12 (green) and PKM2 (red) in OPLL tissue. Scale bar = 30 μm. **H**, **I** Quantitative PCR analysis of ADAM12L and ADAM12S mRNA levels in PLL and OPLL cells. *n* = 3. **J** Quantitative PCR analysis of the expression levels of the ADAM12L and ADAM12S isoforms in OPLL cells. *n* = 3. **K** Western blot analysis of ADAM12 protein levels in PLL and OPLL cells. *n* = 3. **L** ELISA analysis of ADAM12 protein levels in PLL and OPLL ligament cells supernatant. *n* = 5. Data are presented as mean ± standard deviation.

Having observed the association between glycolysis and OPLL, we sought to identify potential upstream regulatory genes. We performed a PPI network analysis of the DEGs from our bulk RNA-seq data. Integration of five network centrality algorithms (DMNC, MCC, MNC, EPC, and Degree) pinpointed nine hub genes, among which *ADAM12* emerged as the most significantly upregulated transcript in OPLL tissues and was identified as a top candidate, particularly by the DMNC algorithm (Fig. 4A, B). Intriguingly, transcriptome-based metabolic flux analysis revealed a strong positive correlation between *ADAM12* expression and glycolytic activity in these tissues (Fig. 4C).

We next validated this association at cellular resolution. Interrogation of our scRNA-seq data confirmed that *ADAM12* was predominantly expressed within the ligament cell compartment of the posterior longitudinal ligament (Fig. 4D, Fig. S3A) and confirmed this by localization with vimentin (Fig. S3C), with a significant positive correlation with glycolytic pathway activity (Fig. 4E). ADAM12 protein was significantly upregulated in human OPLL tissues (Fig. 4F, Fig. S3B) and co-localized with the glycolytic marker PKM2 in ossified lesions (Fig. 4G). Compared to PLL cells, OPLL cells exhibited a significant upregulation of the short isoform (ADAM12S), whereas the long isoform (ADAM12L) remained unchanged (Fig. 4H, I). Meanwhile, the expression of ADAM12S was markedly higher than that of ADAM12L within OPLL cells at mRNA levels (Fig. 4J). Consistently, Western blot analysis using an antibody capable of detecting both isoforms revealed that ADAM12S protein was significantly upregulated in OPLL cells, whereas ADAM12L was barely detectable (Fig. 4K, Fig. S3D). Furthermore, consistent with the secretory nature of the short isoform, ELISA analysis revealed that ADAM12 levels were also significantly elevated in the supernatants of OPLL cells (Fig. 4L). For brevity and clarity, ADAM12 refers to the ADAM12S isoform in the subsequent text and figures unless otherwise specified. We also conducted preliminary investigations, finding that *ADAM12* expression could be induced by inflammatory factors such as IL-6, IL-1β, and TNFα (Fig. S3E).

### ADAM12 accelerates glycolytic flux and lactate production in ligament cells

**Fig. 5:**
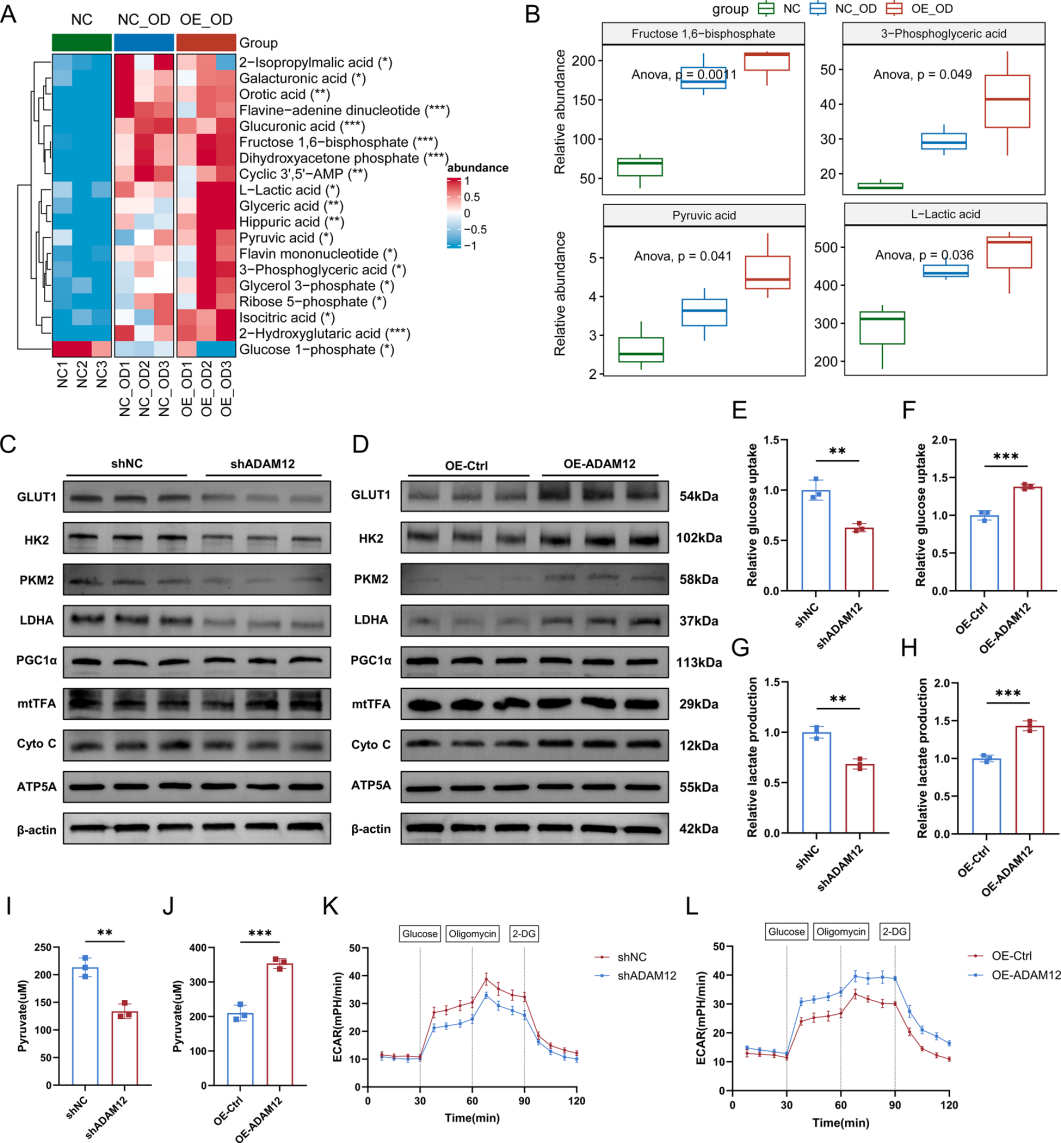
ADAM12 promotes glycolytic flux in ligament cells. **A** Heatmap showing the relative abundance of central carbon metabolites in control (NC), osteogenically differentiated (NC-OD), and ADAM12-overexpressing osteogenically differentiated (OE-OD) ligament cells. *n* = 3. **B** Box plots quantifying the levels of key glycolytic intermediates (fructose-1,6-bisphosphate, 3-phosphoglycerate, pyruvate, and lactate) from the metabolomics data. *n* = 3. **C**, **D** Western blot analysis of glycolytic markers (GLUT1, HK2, PKM2, LDHA) and mitochondrial markers (PGC1α, mtTFA, ATP5A, cytochrome c) in ligament cells after ADAM12 knockdown and overexpression. *n* = 3. **E**, **F** Glucose uptake capacity in ligament cells after ADAM12 knockdown and overexpression. *n* = 3. **G**, **H** Lactate production in ligament cells after ADAM12 knockdown and overexpression. *n* = 3. **I**, **J** Intracellular pyruvate content in ligament cells after ADAM12 knockdown and overexpression. *n* = 3. **K**, **L** ECAR measured by Seahorse XF Analyzer in ligament cells after ADAM12 knockdown and overexpression. *n* = 3. Data are presented as mean ± standard deviation.

To assess the functional role of ADAM12 in glycolysis, we engineered lentiviruses to manipulate its expression in ligament cells. Lentiviruses were used at an MOI of 40, confirmed by GFP visualization (Fig. S3F, G). Stable cell lines with ADAM12 knockdown and overexpression were generated and assessed using qPCR, western blotting, and ELISA (Fig. S3H–K). We performed targeted central carbon metabolomics on control (NC), osteogenically differentiated (NC_OD), and ADAM12-overexpressing (OE_OD) ligament cells. Unsupervised clustering of 56 metabolites revealed distinct metabolic profiles, with osteogenic differentiation inducing marked alterations (Fig. 5A). ADAM12 overexpression further amplified this shift, significantly elevating the levels of key glycolytic intermediates, including fructose-1,6-bisphosphate, 3-phosphoglycerate, pyruvate, and the end-product lactate (Fig. 5A, B).

We next examined the effect of ADAM12 modulation on glycolytic protein expression. Knockdown of ADAM12 decreased, while its overexpression increased, the protein levels of key glycolytic components, including GLUT1, HK2, PKM2, and LDHA. In contrast, the expression of markers for mitochondrial biogenesis and function (PGC1α, mtTFA, ATP5A) remained largely unchanged (Fig. 5C, D, Fig. S4A, B). Similarly, no significant changes in mitochondrial membrane potential were observed following ADAM12 modulation, as assessed by TMRE staining (Fig. S4C, D).

ADAM12 knockdown attenuated, whereas its overexpression enhanced, cellular glucose uptake and lactate production (Fig. 5E–H). Accordingly, intracellular pyruvate levels were also modulated by ADAM12 expression (Fig. 5I, J). Finally, real-time metabolic flux analysis confirmed that ADAM12 knockdown suppressed, while its overexpression elevated ECAR, a direct readout of glycolytic activity (Fig. 5K, L).

### ADAM12 promotes osteogenesis through glycolysis-driven lactate production

**Fig. 6:**
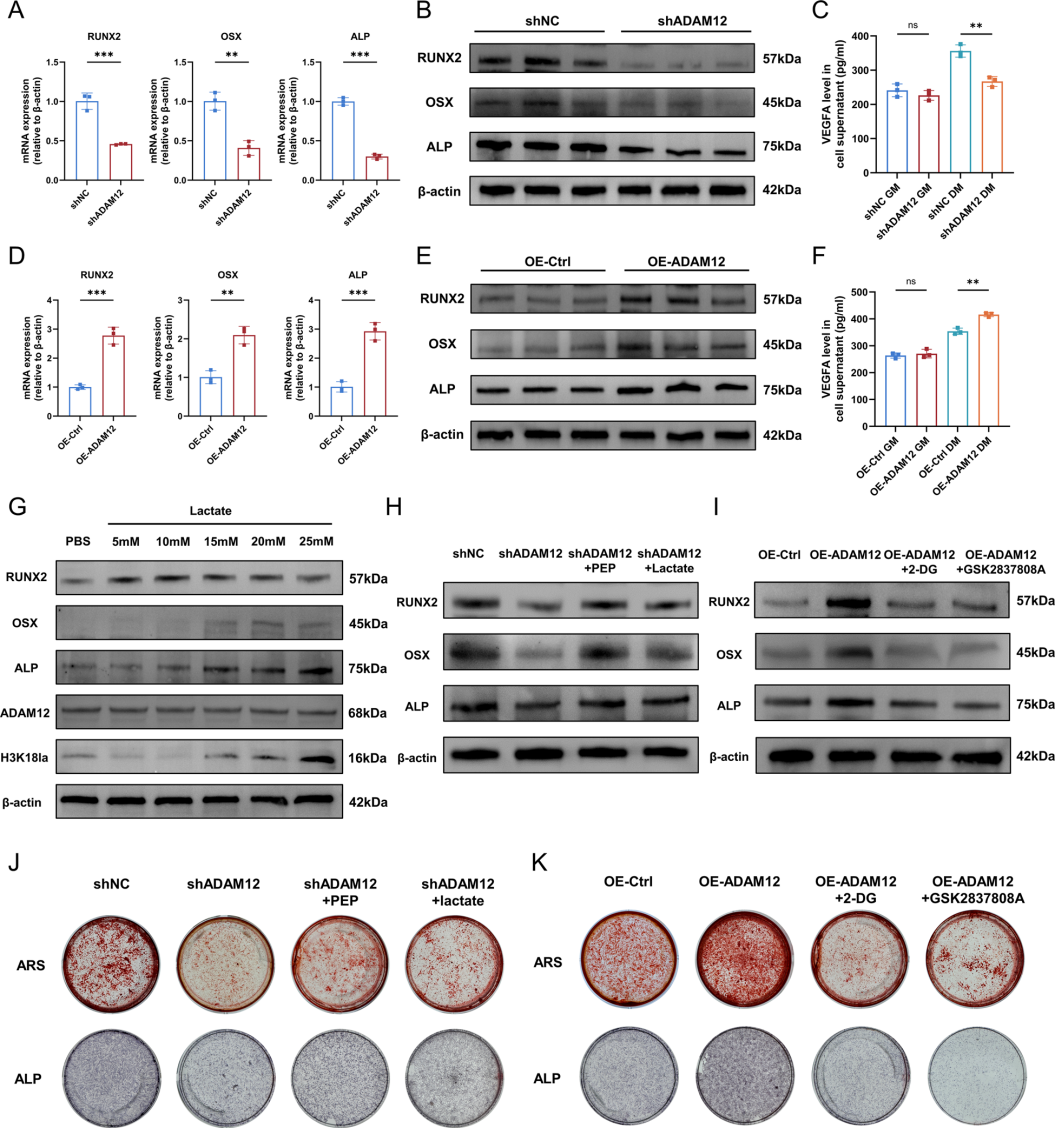
ADAM12 promotes osteogenic differentiation through glycolytic reprogramming and lactate production. **A**, **D** Quantitative PCR analysis of RUNX2, OSX, and ALP mRNA levels in ligament cells after ADAM12 knockdown and overexpression. *n* = 3. **B**, **E** Western blot analysis of RUNX2, OSX, and ALP protein levels after ADAM12 knockdown and overexpression. *n* = 3. **C**, **F** ELISA quantification of VEGFA secretion in cell culture supernatant after ADAM12 knockdown and overexpression. *n* = 3. **G** Western blot analysis of RUNX2, OSX, ALP, ADAM12, and H3K18la in ligament cells treated with increasing concentrations of lactate. **H** Western blot showing recovery of osteogenic marker expression in ADAM12-knockdown cells after treatment with PEP or lactate. **I** Western blot showing suppression of osteogenic markers in ADAM12-overexpressing cells treated with 2-DG or GSK2837808A. **J** ARS and ALP staining showing restoration of mineralization in ADAM12-knockdown cells after PEP or lactate treatment. **K** ARS and ALP staining showing suppression of enhanced mineralization in ADAM12-overexpressing cells after 2-DG or GSK2837808A treatment. Data are presented as mean ± standard deviation.

We next investigated the functional consequence of ADAM12-driven glycolysis on the osteogenic differentiation of ligament cells. Knockdown of ADAM12 significantly reduced, while its overexpression enhanced, the expression of the key osteogenic transcription factors RUNX2 and OSX, and the early differentiation marker ALP, at both the mRNA and protein levels (Fig. 6A, B, D, E, Fig. S5A, B). Consistent with the role of glycolysis in lactate production, supplementing the culture medium with exogenous lactate robustly upregulated osteogenic markers (RUNX2, OSX, ALP) and increased histone H3K18 lactylation (H3K18la) levels, without altering ADAM12 expression (Fig. 6G, Fig. S5C).

In rescue experiments, the impaired osteogenic differentiation caused by ADAM12 knockdown was effectively restored by the addition of phosphoenolpyruvate (PEP, a glycolytic intermediate) or lactate, as evidenced by the recovery of osteogenic marker expression and the restoration of matrix mineralization (Fig. 6H, J, Fig. S5D). Conversely, the enhanced osteogenesis driven by ADAM12 overexpression was blunted by the glycolysis inhibitor 2-DG or the lactate dehydrogenase inhibitor GSK2837808A, which blocks lactate production (Fig. 6I, K, Fig. S5E).

We also assessed the secretion and expression of angiogenesis-related factors. 2-DG significantly inhibited VEGFA secretion by ligament cells (Fig. S6A). ADAM12 knockdown under osteogenic conditions significantly reduced the secretion of VEGFA, whereas ADAM12 overexpression increased it (Fig. 6C, F). ADAM12 also could promote the expression of *VEGFA* and *FGF2*, as well as inhibit the expression of *THBS1* and *TIMP2* (Fig. S6C, D).

### ADAM12 activates the IGF1 signaling pathway to drive glycolytic reprogramming

**Fig. 7:**
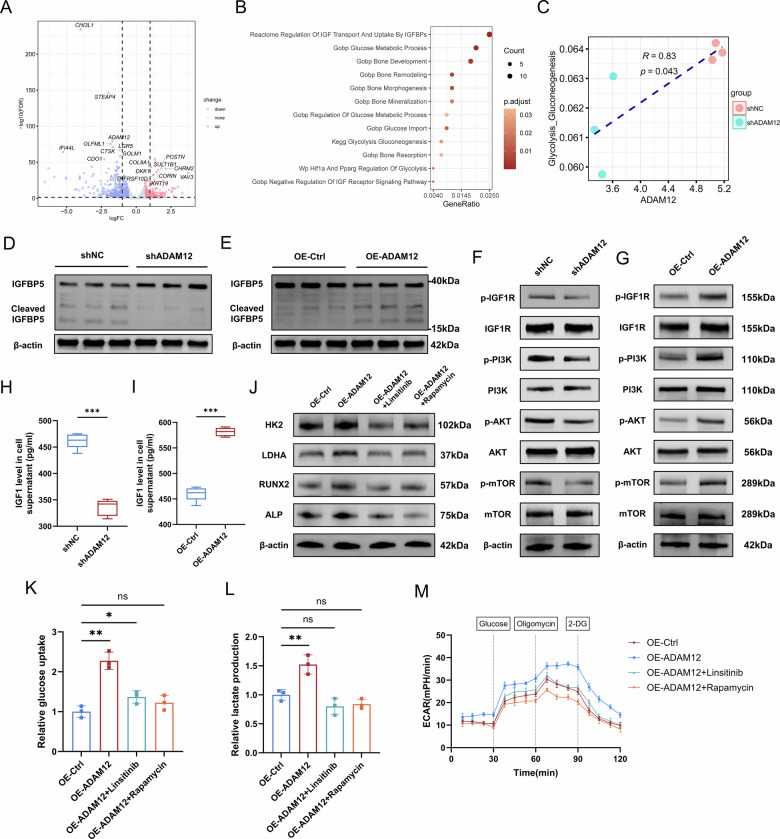
ADAM12 regulates glycolytic reprogramming through IGF1 pathway activation. **A** Volcano plot of DEGs in ligament cells following ADAM12 knockdown. **B** Representative enriched pathways from GO, KEGG, and REACTOME analysis of genes altered by ADAM12 knockdown. **C** Correlation analysis between ADAM12 expression and glycolysis pathway activity from transcriptomic data. **D**, **E** Western blot analysis of IGFBP5 and cleaved IGFBP5 protein levels after ADAM12 knockdown and overexpression. *n* = 3. **F**, **G** Western blot analysis of total and phosphorylated levels of IGF1R, PI3K, AKT, and mTOR proteins after ADAM12 knockdown and overexpression. *n* = 3. **H**, **I** IGF1 concentration in cell culture supernatant measured by ELISA after ADAM12 knockdown and overexpression. *n* = 5. **J** Western blot analysis of glycolytic enzymes (HK2, LDHA) and osteogenic markers (RUNX2, ALP) in ADAM12-overexpressing cells treated with Linsitinib or Rapamycin. *n* = 3. **K**, **L** Glucose uptake and lactate production in ADAM12-overexpressing cells treated with Linsitinib or Rapamycin. *n* = 3. **M** ECAR measurements in ADAM12-overexpressing cells treated with Linsitinib or Rapamycin. *n* = 3. Data are presented as mean ± standard deviation.

To elucidate the mechanism by which ADAM12 enhances glycolysis, we performed transcriptome sequencing of ADAM12-knockdown ligament cells. Pathway enrichment analysis of the resulting differentially expressed genes revealed significant alterations in pathways related to glucose metabolism and bone development, with a pronounced enrichment in the IGF signaling pathway (Fig. 7A, B). Additionally, a positive correlation between ADAM12 and glycolysis was also confirmed at the high-throughput sequencing level (Fig. 7C). This finding was consistent with the established role of ADAM12 in cleaving IGF-binding proteins (IGFBPs) [23]. We therefore hypothesized that ADAM12 regulates glycolysis through IGF1 pathway activation.

We confirmed that neither ADAM12 knockdown nor overexpression significantly altered the mRNA levels of *IGFBP3* or *IGFBP5* (Fig. S7A, B). Despite this transcriptional stability, we found that ADAM12 knockdown resulted in the accumulation of full-length IGFBP5 and a reduction in its cleaved fragments. Conversely, ADAM12 overexpression leads to decreased full-length protein and increased cleaved fragments (Fig. 7D, E). In contrast, ADAM12 modulation had no effect on either the full-length or cleaved forms of IGFBP3 (Fig. S7C, D). Notably, the levels of cleaved IGFBP3 fragments were negligible, suggesting that IGFBP5, rather than IGFBP3, serves as the primary functional IGFBP target of ADAM12 in OPLL cells. Accordingly, ADAM12 knockdown significantly suppressed the phosphorylation of IGF1R, PI3K, AKT, and mTOR, whereas ADAM12 overexpression markedly enhanced the activation of these key signaling effectors (Fig. 7F, G, Fig. S7E, F). Concordantly, IGF1 levels in the cell culture supernatant decreased upon ADAM12 knockdown and increased upon its overexpression (Fig. 7H, I). To directly validate the regulatory role of IGF1 on glycolysis, we treated ligament cells with exogenous IGF1. Western blot analysis revealed that IGF1 stimulation significantly upregulated the protein levels of key glycolytic components, including GLUT1, HK2, and LDHA (Fig. S7G). Functionally, this molecular change was accompanied by a marked increase in both glucose uptake and lactate production (Fig. S7H, I).

To determine whether the IGF1 pathway mediates ADAM12’s metabolic effects, we treated ADAM12-overexpressing cells with the IGF1R inhibitor Linsitinib or the mTOR inhibitor Rapamycin. Both inhibitors effectively blocked the ADAM12-induced upregulation of glycolytic enzymes (HK2, LDHA) and osteogenic markers (RUNX2, OSX) (Fig. 7J, Fig. S7G). Furthermore, the enhanced glucose uptake, lactate production, and elevated ECAR caused by ADAM12 overexpression were all suppressed by Linsitinib and Rapamycin (Fig. 7K–M).

### The metalloproteinase activity of ADAM12 is indispensable for IGF1 activation and glycolysis

To determine whether the metabolic regulatory function of ADAM12 depends on its enzymatic activity, we generated a catalytically inactive mutant, OE-ADAM12^ΔE351Q^. qPCR, Western blot, and ELISA analyses confirmed that the expression levels of ADAM12 in OE-ADAM12^ΔE351Q^ cells were comparable to those in wild-type OE-ADAM12 cells, ruling out dosage effects (Fig. S8A–C). However, while overexpression of wild-type ADAM12 significantly elevated IGF1 levels in the culture supernatant, this effect was substantially attenuated in the OE-ADAM12^ΔE351Q^ group (Fig. S8D). Consistently, the ability to promote glycolysis was also compromised by the mutation; the robust increases in glucose uptake and lactate production observed in OE-ADAM12 cells were significantly blunted in OE-ADAM12^ΔE351Q^ cells (Fig. S8E, F). Parallelly, Western blot analysis revealed that the upregulation of key glycolytic enzymes (GLUT1, HK2, LDHA) induced by wild-type ADAM12 was significantly abrogated in the mutant group (Fig. S8G).

### In vivo targeting of the ADAM12-IGF1-glycolysis axis suppresses ossification

**Fig. 8:**
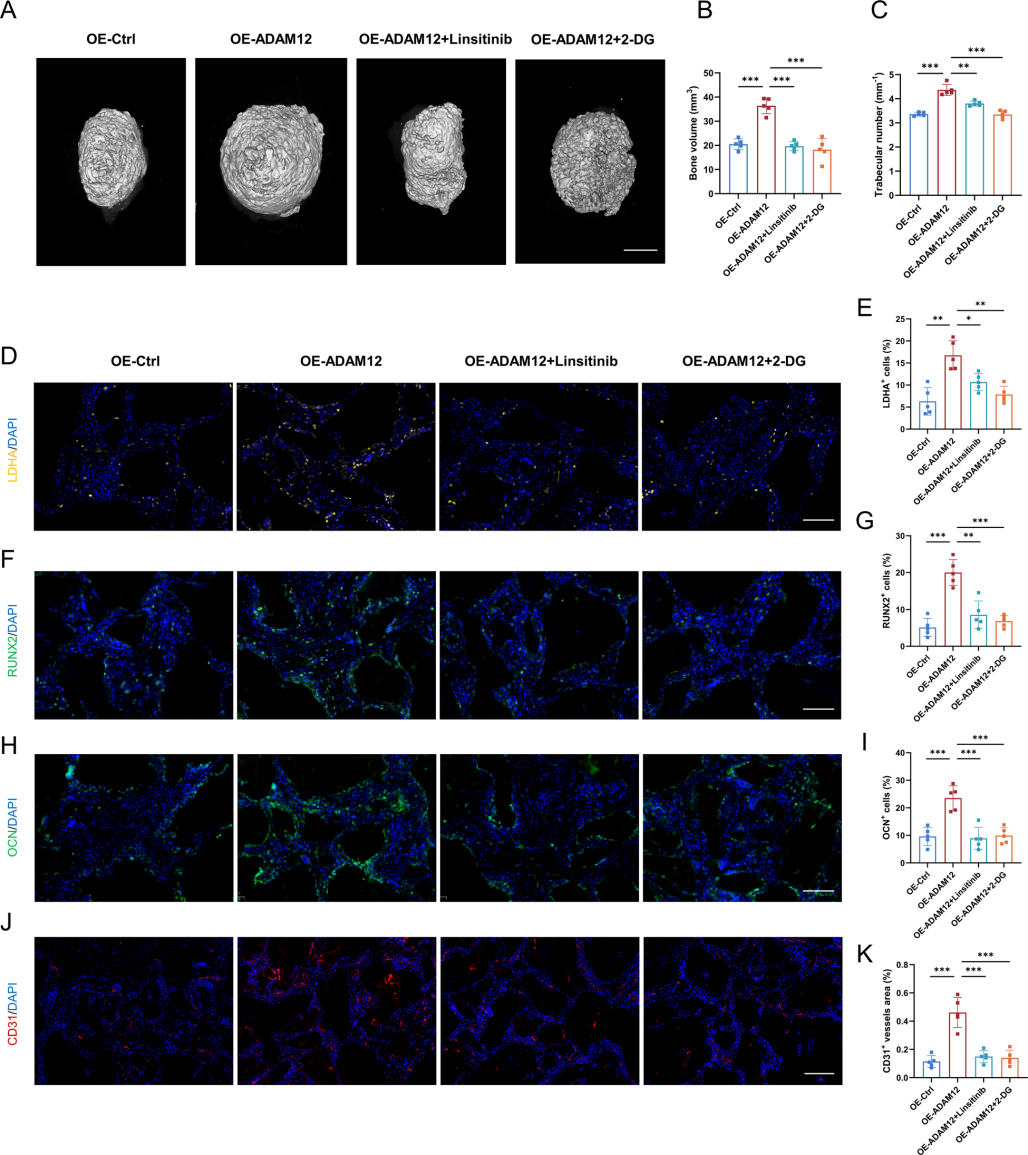
Targeting the ADAM12-IGF1-glycolysis axis suppresses heterotopic ossification in vivo*.* **A** Representative micro-CT images of ectopic bone formation in nude mice after subcutaneous implantation with ligament cells from the indicated treatment groups: control (OE-Ctrl), ADAM12-overexpressing (OE-ADAM12), and ADAM12-overexpressing cells treated with Linsitinib (OE-ADAM12+Linsitinib) or 2-DG (OE-ADAM12 + 2-DG). Scale bar = 1 mm. **B**, **C** Quantitative analysis of bone volume and trabecular number from micro-CT data. *n* = 5. **D**, **E** Representative immunofluorescence images and quantification of LDHA^+^ positive cells within the ectopic bone masses. Scale bar = 100 µm, *n* = 5. **F**, **G** Representative immunofluorescence images and quantification of RUNX2^+^ cells. Scale bar = 100 µm, *n* = 5. **H**, **I** Representative immunofluorescence images and quantification of OCN^+^ cells. Scale bar = 100 µm, *n* = 5. **J**, **K** Representative immunofluorescence images and quantification of CD31^+^ positive vascular areas. Scale bar = 200 µm, *n* = 5. Data are presented as mean ± standard deviation.

To evaluate the effects of targeting the ADAM12-IGF1-glycolysis axis in vivo, we established an in vivo ectopic ossification model by subcutaneously implanting ligament cells with modulated ADAM12 expression in nude mice. Micro-CT analysis after 8 weeks revealed that ADAM12 overexpression (OE-ADAM12) significantly increased bone volume and trabecular number compared to the control (OE-Ctrl) (Fig. 8A–C). Crucially, this pro-ossific effect was substantially attenuated by co-treatment with either the IGF1R inhibitor Linsitinib or the glycolysis inhibitor 2-DG (OE-ADAM12 + Linsitinib/2-DG) (Fig. 8A–C).

Immunofluorescence analysis of the explanted tissues showed that the OE-ADAM12 group exhibited a significant increase in LDHA⁺ cells, which was reversed by Linsitinib and 2-DG treatment (Fig. 8D, E), confirming the in vivo link between ADAM12 and glycolytic activity. Accordingly, the populations of RUNX2⁺ osteoprogenitor cells and OCN⁺ mature osteoblasts were expanded by ADAM12 overexpression and reduced upon pathway inhibition (Fig. 8F–I). Furthermore, ADAM12 overexpression promoted CD31⁺ vascular area within the ossified tissue, an effect that was also suppressed by Linsitinib and 2-DG (Fig. 8J, K).

## Discussion

In this study, we uncover a fundamental metabolic vulnerability in OPLL and establish the ADAM12-IGF1-glycolysis axis as a central engine of pathological ossification. By integrating single-cell metabolic profiling with rigorous functional validation, we demonstrate that the transition to a glycolytic phenotype is not merely a passive adaptation to energy demand, but an active, indispensable driver of lineage commitment. We conceptually advance the field by identifying lactate as a novel epigenetic signal that translates metabolic flux into osteogenic gene expression. Furthermore, we distinguish the secreted isoform ADAM12 as the master upstream regulator, validating that pharmacological blockade of this ADAM12-driven axis effectively arrests ectopic bone formation in vivo.

OPLL is a multifactorial spinal disorder that can be idiopathic or secondary to systemic diseases, often progressing from a clinically silent stage to cause severe neurological deficits such as quadriplegia [1, 26]. Surgical decompression, the mainstay for severe cases, carries substantial risks and fails to halt ossification progression, highlighting an urgent need for mechanism-based therapies [27]. Despite known associations with genetic, mechanical, and metabolic factors [2, 28], a unifying pathogenic driver has remained elusive. Our study addresses this gap by identifying pathologic metabolic reprogramming as a central hub in OPLL pathogenesis.

The cellular origin of OPLL has been a subject of ongoing investigation. Our single-cell transcriptomic data robustly identify PRG4⁺ ligament progenitor cells as a primary source of osteoblasts in OPLL. This finding directly supports and extends recent work implicating PRG4⁺ progenitors in aberrant tendon ossification [29, 30], confirming their broader role in pathological fibro-osseous disorders beyond the tendon niche. The metabolic phenotype (a marked enhancement of glycolysis coupled with suppression of oxidative phosphorylation) we observed during their differentiation aligns with the recognized anabolic demands of bone matrix synthesis [31] and mirrors the metabolic strategy employed by physiological osteoblasts, which prioritize glycolytic flux for biosynthetic precursor and ATP production [19, 20, 32, 33]. However, while aerobic glycolysis is established in physiological osteogenesis [34], its specific role and regulatory mechanisms in pathological ligament ossification have not been previously investigated. Our work now positions this metabolic program as a central pathological mechanism in OPLL. The potent suppression of ossification by 2-DG in our mouse model not only validates this mechanistic link but also highlights a metabolic vulnerability that could be therapeutically targeted.

The lack of significant change in ROS levels between OPLL and PLL cells suggests a tightly regulated redox environment, possibly to avoid the inhibitory effects of excessive ROS on osteogenic differentiation [35, 36]. Critically, this observation further distinguishes the metabolic shift in OPLL from a mere stress response; instead, it underscores that the glycolytic reprogramming we identified is primarily geared toward supporting anabolic biosynthesis, rather than being a secondary consequence of oxidative stress.

Our study redefines the biological significance of ADAM12, specifically distinguishing the secreted isoform ADAM12S, as a central pathogenic driver that orchestrates metabolic reprogramming in OPLL. While prior work links ADAM12 to metabolism in cancer via HK1 [25] and to IGF1 signaling in skeletal development [24, 37–39], and notes general metalloproteinase elevation in ligament degeneration [40], the causal role of ADAM12 in OPLL and its direct mechanistic link to the glycolytic switch were entirely unknown. Here, we identify ADAM12 as a critical upstream metabolic driver. Unlike a simple upregulation, the secretion of ADAM12 enables the proteolytic liberation of IGF1 from IGFBP5, thereby enhancing local growth factor bioavailability. This event triggers the PI3K-AKT-mTOR cascade, effectively enforcing the glycolytic state required for ossification. Thus, our findings connect extracellular matrix remodeling directly to intracellular metabolic rewiring, establishing a linear, targetable axis specific to OPLL pathogenesis.

Our study defines a novel pathogenic mechanism in OPLL by identifying lactate not merely as a metabolic byproduct, but as the essential signaling effector that translates ADAM12-driven glycolysis into osteogenic commitment. While lactate-driven histone lactylation is an emerging regulatory paradigm in other contexts [41, 42], including physiological bone homeostasis [13, 14], its functional role and upstream regulation within a specific pathological ossification process were unknown. We demonstrate that exogenous lactate fully rescues osteogenesis in ADAM12-deficient cells, accompanied by elevated histone H3K18 lactylation. This confirms that ADAM12 drives osteogenesis through glycolysis-derived lactate, illustrating how metabolic reprogramming actively directs cell fate via epigenetic rewiring.

Beyond fueling osteogenesis through lactate production, which we show can itself promote histone lactylation and osteogenic marker expression, ADAM12-driven glycolysis also orchestrates the pro-angiogenic microenvironment conducive to ossification. The ADAM12-mediated promotion of VEGFA and FGF2, coupled with the suppression of anti-angiogenic factors THBS1 and TIMP2, positions ADAM12 as a central coordinator of the multicellular processes required for ectopic bone formation. This aligns with its reported roles in tumor angiogenesis [43, 44] and underscores the interconnectedness of metabolic reprogramming and vascularization in OPLL pathogenesis.

While our study establishes ADAM12 as a central driver, the broader protease network in OPLL, including other upregulated factors like MMP1, may also contribute to IGF signaling and tissue remodeling. Our functional data define the ADAM12/IGF1 axis as a critical pathogenic module, though in vivo cooperation with other proteases could enhance ossification robustness. Future studies should explore the interplay between ADAM12 and other upregulated proteases to fully decipher the dysregulated microenvironment in OPLL.

We acknowledge several limitations in our study. First, while our animal model provides valuable insights into the ossification process, it does not fully recapitulate the slow, progressive nature of human OPLL within the complex biomechanical environment of the spine. Second, although we have established the crucial role of ADAM12, the upstream mechanisms triggering its upregulation warrant further investigation. Additionally, while our data indicate that ADAM12 acts as an upstream initiator unaffected by acute lactate exposure, the possibility of feedback regulation from downstream metabolic pathways cannot be ruled out. Finally, the clinical translation of these findings will depend on the future development of specific ADAM12 inhibitors and their rigorous evaluation in disease-relevant models.

In conclusion, we have uncovered the ADAM12-IGF1-glycolysis axis as a central regulatory mechanism in OPLL. This pathway integrates a key metalloproteinase, a classic anabolic signaling cascade, and a fundamental metabolic program to fuel the pathological ossification of the spinal ligament. The metabolic dependency of OPLL on glycolysis represents a critical vulnerability that can be therapeutically exploited. Our findings not only deepen the molecular understanding of OPLL pathogenesis but also position ADAM12 and the glycolytic pathway as promising therapeutic targets for a debilitating condition that currently lacks effective pharmacological treatments.

## Methods

### Patient cohorts and tissue acquisition

Human posterior longitudinal ligament tissues were obtained from patients diagnosed with ossification of the posterior longitudinal ligament (OPLL, *n* = 10) or non-OPLL degenerative conditions (cervical spondylotic myelopathy or radiculopathy, *n* = 10). Diagnosis was confirmed through a combination of clinical symptom assessment, radiological evaluations, and direct intraoperative observation. All patients underwent a standard anterior cervical discectomy and fusion procedure. Immediately after resection, tissue samples were rinsed thoroughly with sterile saline, placed in a sterile container with saline-moistened gauze, and transported to the laboratory on ice within 1 h. Detailed patient demographics and clinical information are summarized in Supplementary Table S1. This study was approved by the Ethics Committee of Shanghai Changzheng Hospital, and written informed consent was obtained from all participants prior to surgery.

### Primary cell culture and characterization

Upon receipt, the ligament tissues were rinsed twice in phosphate-buffered saline (PBS) containing 1% penicillin/streptomycin to minimize microbial contamination. The tissues were then meticulously dissected to remove any adherent blood vessels or adipose tissue and cut into approximately 1 mm³ explants. These explants were evenly distributed in 10 cm culture dishes and allowed to adhere for 10–15 min before the careful addition of mesenchymal stem cell basal medium (Dakewe, China) supplemented with 10% fetal bovine serum (Gibco, USA) and 1% penicillin/streptomycin (Gibco, USA). The cultures were maintained at 37 °C in a humidified atmosphere of 5% CO₂. The medium was replaced every 3 days. Outgrowth cells from the explants were confirmed to be free of mycoplasma contamination and were passaged upon reaching 70–80% confluence using 0.25% Trypsin-EDTA (Gibco, USA) for approximately 1 min. To maintain a stable and responsive phenotype, cells from passages 2 to 5 were used for all subsequent experiments. The ligament-derived identity of these cells was confirmed by their spindle-shaped, fibroblast-like morphology and positive immunostaining for the ligament progenitor marker PRG4.

### Osteogenic differentiation

To induce osteogenic differentiation, ligament cells derived from either OPLL or control PLL tissues were seeded in 24-well plates at a density of 2 × 10^4^ cells per well. After allowing cell attachment for 24 h in standard growth medium, the culture medium was aspirated and replaced with a commercial osteogenic induction medium (Oricell, China). This induction medium is formulated with supplementary components including dexamethasone, ascorbic acid, and β-glycerophosphate. The osteogenic induction medium was refreshed every 3 days throughout the induction period.

### Alkaline phosphatase (ALP) staining

Cells were seeded in 24-well plates at a density of 2 × 10^4^ cells per well and induced for 7 days, then fixed with 4% paraformaldehyde and stained using a BCIP/NBT-based ALP staining kit (Beyotime, P0321S) according to the manufacturer’s instructions, to detect early osteogenic activity.

### Alizarin Red S (ARS) staining

To detect late-stage mineralization and calcium deposition, cells were seeded in 24-well plates at a density of 2 × 10^4^ cells per well and induced for 14 days, then fixed with 4% paraformaldehyde and stained with a 2% Alizarin Red S solution (Oricell, ALIR-10001). The stained mineralized nodules were visualized under a light microscope.

### Glucose uptake and lactate production measurements

For metabolite measurement assays, ligament cells were seeded in 24-well plates at a density of 2 × 10^4^ cells per well. Following designated treatments, cell culture supernatants were collected and centrifuged at 1000 × *g* for 5 min to remove cellular debris. The cleared supernatants were then analyzed using commercial colorimetric assay kits according to the manufacturers’ protocols. Specifically, glucose concentration was determined using a Glucose Assay Kit (Nanjing Jiancheng Bioengineering Institute, F006-1-1) based on the glucose oxidase-peroxidase method, with absorbance measured at 505 nm. Lactate concentration was measured using a Lactate Assay Kit (Nanjing Jiancheng Bioengineering Institute, A019-2-1), with absorbance read at 530 nm. In addition, piruvate concentration was measured using a Piruvate Assay Kit (Nanjing Jiancheng Bioengineering Institute, A081-1-1), with absorbance read at 505 nm.

### Measurement of intracellular reactive oxygen species (ROS)

Intracellular ROS levels were detected using a commercial assay kit (Solarbio, CA142) based on the fluorescent probe DCFH-DA. Ligament cells were seeded in 24-well plates at a density of 2 × 10^4^ cells per well. After treatment, cells were incubated with 10 μM DCFH-DA in serum-free medium at 37 °C for 20 min in the dark. After washing with PBS to remove excess probe, the fluorescence of the oxidized product was captured using a fluorescence microscope.

### Mitochondria transmembrane potential assay

Mitochondrial membrane potential was assessed using the TMRE assay kit (Beyotime, C2001S). Ligament cells were seeded in 6-well plates at a density of 1 × 10^5^ cells per well. Following the designated treatments, cells were washed with PBS and incubated with TMRE working solution for 20 min in the dark. After incubation, the cells were washed twice with PBS to remove excess dye. TMRE is a cell-permeant, positively-charged dye that accumulates in active mitochondria due to their relative negative charge; therefore, sequestered TMRE fluorescence intensity is directly proportional to the mitochondrial membrane potential. Fluorescence images were captured using a fluorescence microscope. The relative fluorescence intensity was quantified using ImageJ software.

### Measurement of ECAR and OCR

Extracellular acidification rate (ECAR) and oxygen consumption rate (OCR) were measured using XF96 Extracellular Flux Analyzer (Agilent Technologies). Briefly, a poly-L-lysine-coated XF96 microplate (Agilent Technologies) was seeded with ligament cells at a density of 1 × 10^4^ cells per well. On the day of measurement, ligament cells were washed twice with XF medium and incubated for 1 h in a CO_2_-free incubator at 37 °C before loading. Glucose (10 mM), oligomycin (2 μM), FCCP (1 μM), antimycin A (0.5 μM), rotenone (0.5 μM), and 2-deoxy-glucose (50 mM) were used to measure ECAR (mpH/min) and OCR (pmol/min). Data were normalized to cell number.

### Lentiviral construction and transfection

Given that the secreted short isoform (ADAM12S) was identified as the predominant variant upregulated in OPLL cells, lentiviral vectors were constructed specifically targeting this isoform. Lentiviral vectors for ADAM12S overexpression (OE-ADAM12) and knockdown (shADAM12), along with their respective controls (OE-Ctrl, shNC), were constructed by Obio Technology (Shanghai, China). To investigate whether the function of ADAM12S depends on its proteolytic activity, we generated a catalytically inactive ADAM12 mutant, ADAM12S^ΔE351Q^ (OE-ADAM12^ΔE351Q^) from Obio Technology. Briefly, guanine (1059) was substituted for cytosine, resulting in a translated protein consisting of a glutamine (Q) at amino acid 351 instead of a glutamate (E), thus rendering the protein catalytically inactive [45]. Ligament cells were seeded in 6-well plates at a density of 1 × 10⁵ cells per well. After overnight culture, cells were transduced with lentiviruses at a multiplicity of infection (MOI) of 40 in the presence of 5 μg/mL polybrene. After 16–18 h, the virus-containing medium was replaced with fresh growth medium. The efficacy of ADAM12 overexpression or knockdown was confirmed by quantitative PCR and Western blot analysis prior to functional experiments.

### Western blotting

Cells were lysed in RIPA buffer (Epizyme, PC101) supplemented with protease and phosphatase inhibitor cocktail (Epizyme, GRF103) on ice. Lysates were centrifuged at 12,000 × *g* for 15 min at 4 °C, and the supernatant protein concentration was determined using a BCA protein assay kit (Epizyme, ZJ101). Equal amounts of protein were separated by 10% SDS-PAGE and transferred to polyvinylidene fluoride (PVDF) membranes (Millipore, IPVH00010). Membranes were blocked with 5% bovine serum albumin (BSA) in TBST for 1 h at room temperature and subsequently incubated with specific primary antibodies (diluted 1:1000) overnight at 4 °C. After washing, membranes were incubated with HRP-conjugated secondary antibodies (Abclonal, AS014, 1:5000) for 1 h at room temperature. Protein bands were visualized using ECL substrate and imaged with a ChemiDoc Imaging System (Bio-Rad, USA). Primary antibodies used were as follows: ADAM12 (Proteintech, 14139-1-AP), RUNX2 (Abclonal, A11753), OSX (Abclonal, A18699), ALP (Zenbio, 381009), HK2 (Zenbio, R24552), PKM2 (Zenbio, R381318), GLUT1 (Zenbio, R380464), PDK1 (Zenbio, R381931), LDHA (Zenbio, R22873), cytochrome c (Zenbio, R22867), ATP5A (Zenbio, R381760), PGC1α (Zenbio, R381615), mtTFA (Zenbio, R22594), IGFBP5 (Proteintech, 55205-1-AP), IGFBP3 (Proteintech, 10189-2-AP), IGF1R (Zenbio, R26882), phosphorylated IGF1R (Zenbio, R24720), PI3K (Zenbio, R25368), phosphorylated PI3K (Zenbio, 341468), AKT (Zenbio, 342529), phosphorylated AKT (Zenbio, 310021), mTOR (Zenbio, R380411), phosphorylated mTOR (Zenbio, R25033), and β-actin (Zenbio, R380624). Full and uncropped images of all Western blots are provided in the Supplementary Material.

### Real-time quantitative PCR

Total RNA was extracted using the FastPure Cell/Tissue Total RNA Isolation Kit V2 (Vazyme, RC112-01). cDNA was synthesized from 1 μg RNA using HiScript III All-in-one RT SuperMix (Vazyme, R333-01). qPCR was performed on a QuantStudio 3 system (Thermo Fisher) using ChamQ Universal SYBR qPCR Master Mix (Vazyme, Q711-02). Each 20 μL reaction contained 10 μL Master Mix, 0.8 μL of each primer, and 2 μL cDNA template. The thermal profile was: 95 °C for 30 s, followed by 40 cycles of 95 °C for 10 s and 60 °C for 30 s. Melt curve analysis confirmed amplification specificity. Relative gene expression was calculated using the 2^-ΔΔCt^ method with β-actin for normalization. Primer sequences are listed in Supplementary Table S2.

### Immunofluorescence analysis

For tissue sections, specimens were fixed in 4% paraformaldehyde for 48 h, decalcified in EDTA, and embedded in paraffin. Sections were deparaffinized and subjected to antigen retrieval in citrate buffer. For cells, samples were fixed with 4% paraformaldehyde for 15 min and permeabilized with 0.1% Triton X-100. After blocking with 5% BSA, samples were incubated overnight at 4 °C with primary antibodies, followed by incubation with fluorescently labeled secondary antibodies for 1 h at room temperature. Nuclei were counterstained with DAPI. Images were captured using a fluorescence microscope. The primary antibodies and dilutions used were: ADAM12 (Proteintech, 14139-1-AP, 1:100), RUNX2 (Abclonal, A11753, 1:600), OCN (Proteintech, 23418-1-IG, 1:600), PKM2 (Proteintech, 60268-1-IG, 1:1500), LDHA (Servicebio, GB11342-100, 1:300), CD31 (Servicebio, GB11063-2-50, 1:1500), PRG4 (Abcam, ab28484, 1:200) and Vimentin (Proteintech, 10366-1-AP, 1:200).

### Medium supernatant ADAM12, VEGFA and IGF1 analysis

The concentrations of ADAM12, vascular endothelial growth factor A (VEGFA), and insulin-like growth factor 1 (IGF1) in ligament cell culture supernatants were determined using commercial ELISA kits. Ligament cells were seeded in 24-well plates at a density of 2 × 10^4^ cells per well. Supernatants were collected and centrifuged to remove cellular debris. The concentrations of ADAM12, VEGFA, and IGF1 were quantified using specific human ELISA kits: ADAM12 (Proteintech, KE00058), VEGFA (AiFang, DVE00), and IGF1 (Beyotime, PI488). All assays were performed strictly according to the manufacturers’ protocols. The absorbance was measured using a microplate reader, and analyte concentrations were calculated from the standard curve generated for each assay.

### Bulk RNA-seq analysis

Bulk RNA-seq data from human OPLL and non-OPLL tissues were obtained from BioProject (ID: PRJNA902554). Additionally, transcriptome data from ligament cells transduced with shADAM12 or shNC were generated by Biotree Biotech Co., Ltd. (Shanghai, China). Raw sequencing reads were quality-controlled using Trim Galore (v0.6.6) and FastQC (v0.11.9) to remove adapter sequences and low-quality bases. High-quality reads were aligned to the human reference genome (GRCh38.p13) using HISAT2 (v2.2.1). Gene-level read counts were generated using featureCounts (v2.0.1). Differential expression analysis was performed using the edgeR package in R, with genes showing |log2(fold change)| >1 and *p*-value < 0.05 considered statistically significant. Functional enrichment analysis of Gene Ontology (GO) terms and Kyoto Encyclopedia of Genes and Genomes (KEGG) pathways was performed using the ClusterProfiler package. Protein-protein interaction (PPI) network analysis was conducted using the STRING database and visualized in Cytoscape (v3.8.2), with hub genes identified using the cytoHubba plugin.

### ScRNA-seq analysis

ScRNA-seq data were obtained from BioProject (ID: PRJNA1067846). The raw sequencing data were processed using Cell Ranger (v7.1.0) with the GRCh38 human reference genome. Downstream analysis was performed using the Scanpy (v1.9.3) package [46]. Quality control was applied to remove low-quality cells and doublets using the following thresholds: genes detected per cell between 500 and 8000, unique molecular identifier (UMI) counts between 1000 and 40,000, and mitochondrial gene percentage <15%. Doublets were removed using Scrublet (v0.2.3) [47]. The data were normalized using the sc.pp.normalize_total function and log-transformed using sc.pp.log1p.

Highly variable genes (*n* = 4000) were selected using sc.pp.highly_variable_genes. The data were scaled, and the effects of total counts and mitochondrial percentage were regressed out. Principal component analysis was performed, and batch effects between patients were corrected using BBKNN (v1.3.1) [48]. Cells were clustered using the Leiden algorithm and visualized with UMAP. Cell types were annotated based on canonical marker genes: ligament cells (DCN) and subpopulations (PRG4, COL3A1, IGFBP6, ACAN, BGLAP), endothelial cells (PECAM1), pericytes (RGS5, ACTA2), monocytes (LYZ, S100A8, S100A9), macrophages (CD68, MRC1), T cells (CD3E), B cells (MS4A1), and plasma cells (JCHAIN).

Cell differentiation trajectory was inferred using the sctour (v1.0.0) package [49]. Metabolic activity was quantified at single-cell resolution using the ScMetabolism (v0.2.1) package [50] with the REACTOME metabolic gene sets. The AUCell method was used to calculate metabolic pathway activity scores.

### Targeted metabolomics analysis

Targeted metabolomics profiling of central carbon metabolites was performed by Biotree Biotech Co., Ltd. (Shanghai, China). A total of 56 key metabolites were quantitatively analyzed. Raw data were preprocessed by replacing any missing values with half of the minimum value for the corresponding metabolite. The resulting dataset was then imported into SIMCA software (v18.0.1) for multivariate statistical analysis. Prior to analysis, the data were scaled and log-transformed to reduce heteroscedasticity and the influence of high variance. An unsupervised hierarchical clustering analysis, based on Euclidean distance and complete linkage, was performed and visualized as a heatmap. Differences in metabolite levels between experimental groups were assessed by one-way analysis of variance (ANOVA), with results displayed using box plots.

### OPLL model analysis

To assess the osteogenic capacity of human ligament cells in vivo, a heterotopic ossification model was established. 2 × 10⁵ ligament cells were co-cultured with Bio-Oss Collagen scaffolds (Geistlich, Germany) in osteogenic medium for 2 days. Four-week-old male BALB/c homozygous nude mice (Shanghai Leigen Biotechnology) were randomly divided into experimental groups. For scaffold implantation, mice were anesthetized with isoflurane, and the dorsal skin was shaved and disinfected. A 1-cm longitudinal midline incision was made in the upper dorsal skin. A subcutaneous pocket was carefully created by blunt dissection. One cell-seeded scaffold was inserted into each pocket. The incision was closed with sterile surgical sutures. After 8 weeks, the mice were euthanized, and the implants were harvested for micro-CT analysis and histological examination.

To evaluate the therapeutic potential of glycolysis inhibition on ossification, a BMP2-induced model was utilized. Six-week-old male C57BL/6J mice (Shanghai Leigen Biotechnology) were anesthetized, and a 1 cm longitudinal incision was made on the back. An rhBMP-2-containing biomaterial (Hangzhou Jiuyuan Gene Engineering) was implanted subcutaneously and secured to the back muscles. Starting on postoperative day 2, mice received intraperitoneal injections of 2-deoxy-D-glucose (50 or 150 mg/kg) or PBS vehicle control every 3 days. At 14 days post-implantation, mice were euthanized, and the resulting heterotopic ossification masses were excised for subsequent analysis. Investigators were blinded to group allocation during micro-CT analysis and histological grading. All animal experiments were approved by the Ethics Committee of Shanghai Changzheng Hospital and conducted in accordance with the institutional guidelines.

### Micro-CT analysis

Harvested tissue samples were fixed in 4% paraformaldehyde and scanned using a Venus Micro-CT system (VNC-102, Pingseng Scientific) at 90 kV and 0.09 mA with an isotropic resolution of 10 μm. Three-dimensional reconstruction and quantitative analysis of bone parameters, including bone volume and trabecular number, were performed using the manufacturer’s software (Cruiser, Recon, and Avatar).

### Statistical analysis

All quantitative data are presented as the mean ± standard deviation (SD) from at least three independent biological replicates. Sample sizes were determined based on preliminary experiments and similar studies in the field to ensure adequate statistical power. No samples or animals were excluded from the analysis. Statistical analyses were performed using GraphPad Prism software (Version 9.0). For comparisons between two groups, a two-tailed Student’s *t*-test was applied. For multiple group comparisons, one-way analysis of variance (ANOVA) followed by Tukey’s post-hoc test was used. The normality of data distribution was assessed using the Shapiro-Wilk test. Variance was assumed to be similar between groups based on similar experimental conditions. A *p*-value of less than 0.05 was considered statistically significant (*p < 0.05, **p < 0.01, ***p < 0.001, ns, not significant).

## Supplementary information

Supplementary Figure 1

Supplementary Figure 2

Supplementary Figure 3

Supplementary Figure 4

Supplementary Figure 5

Supplementary Figure 6

Supplementary Figure 7

Supplementary Figure 8

Supplementary Figure legend

Supplementary Table

Full and uncropped western blots

## Data Availability

All data generated or analyzed during this study are included in this published article and its supplementary information files.
