# Supplementary figures and images for: Glycolytic reprogramming mediated by the ADAM12/IGF1 axis promotes ossification of the posterior longitudinal ligament
Source: Cell Death Discov. 2026 Mar 25;12:178. doi: 10.1038/s41420-026-03044-8 (PMC13039164; doi:10.1038/s41420-026-03044-8)

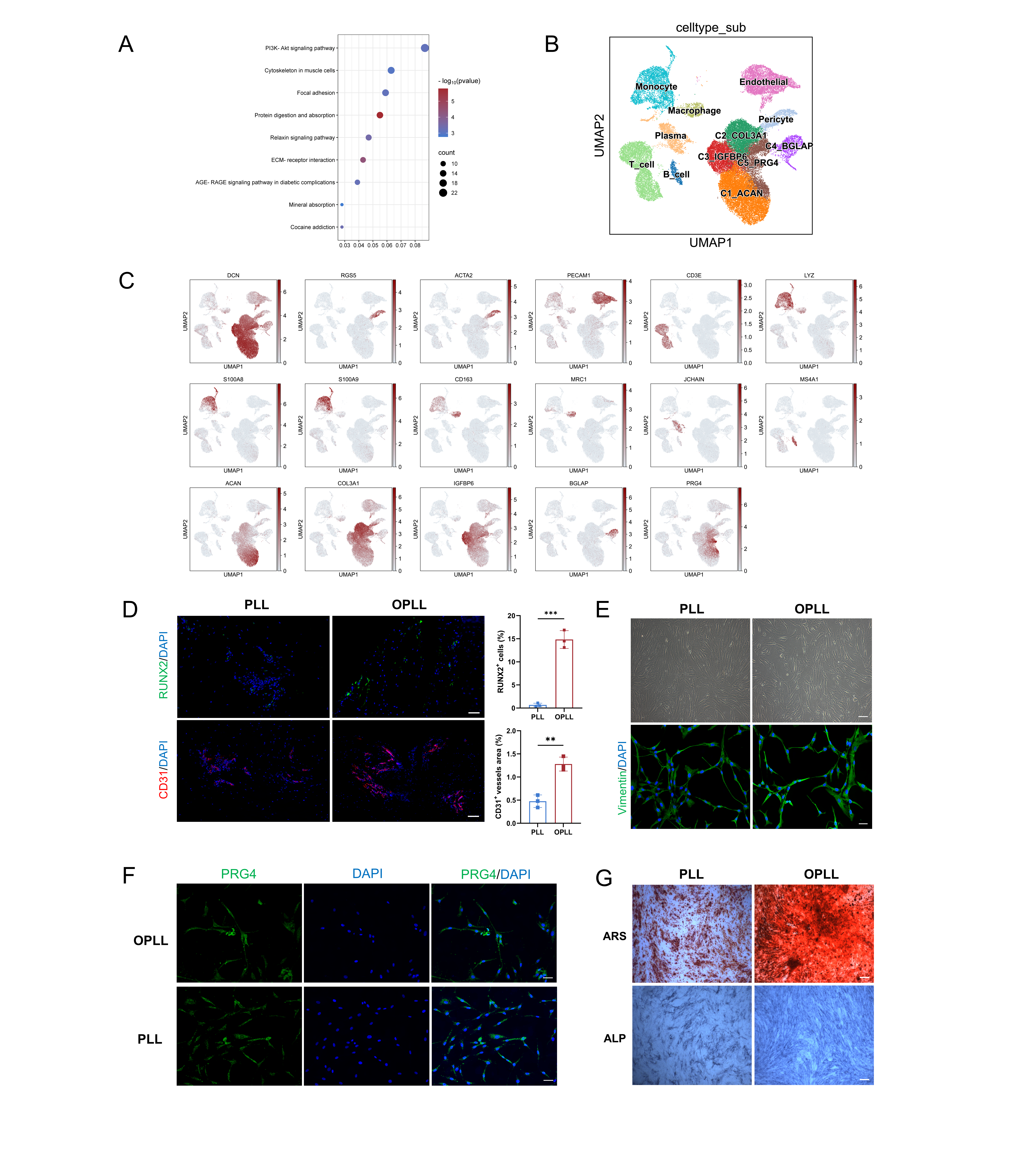

Supplement: Supplementary file 1 — Supplementary Figure 1 [file 41420_2026_3044_MOESM1_ESM.tif]

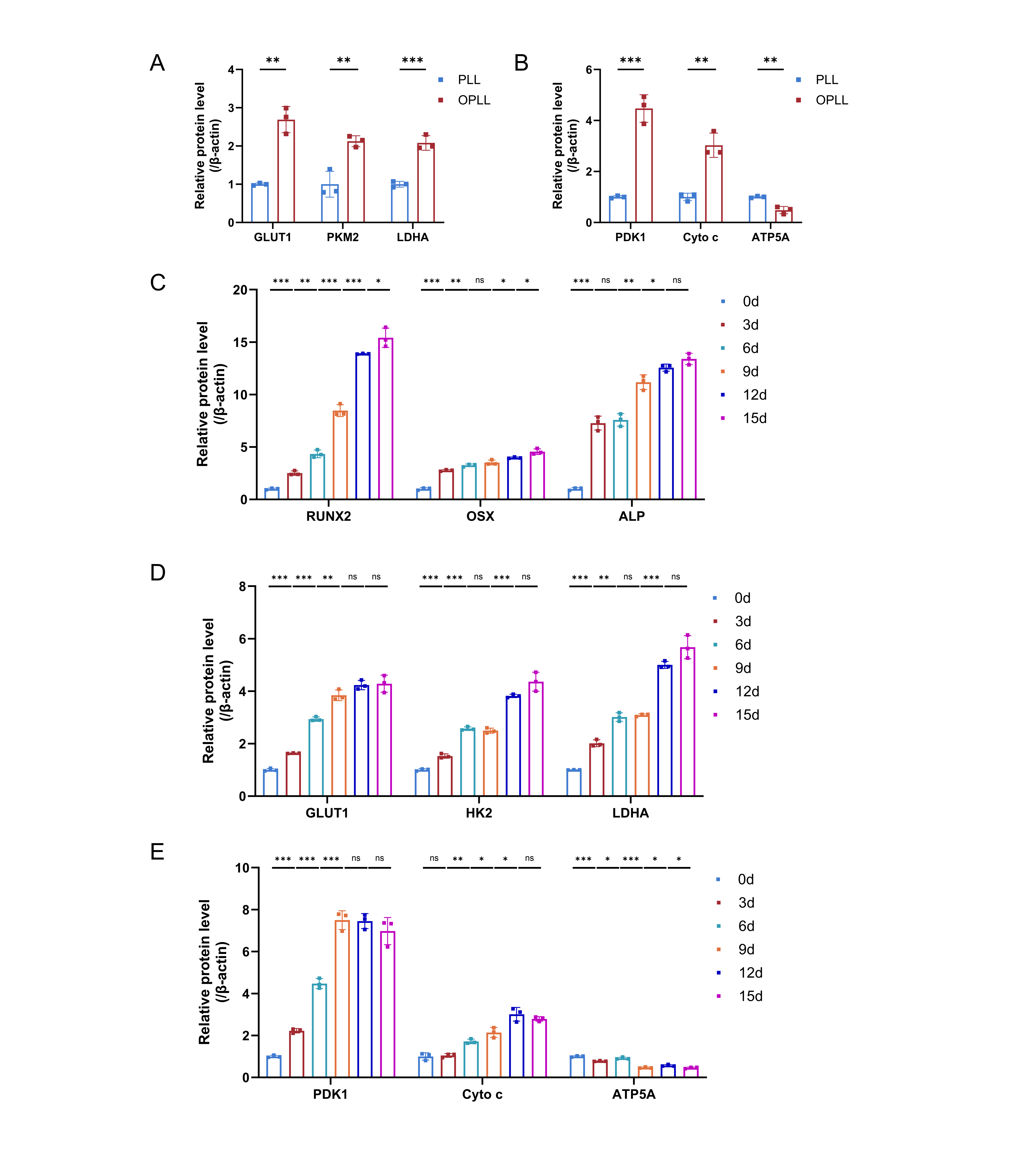

Supplement: Supplementary file 2 — Supplementary Figure 2 [file 41420_2026_3044_MOESM2_ESM.tif]

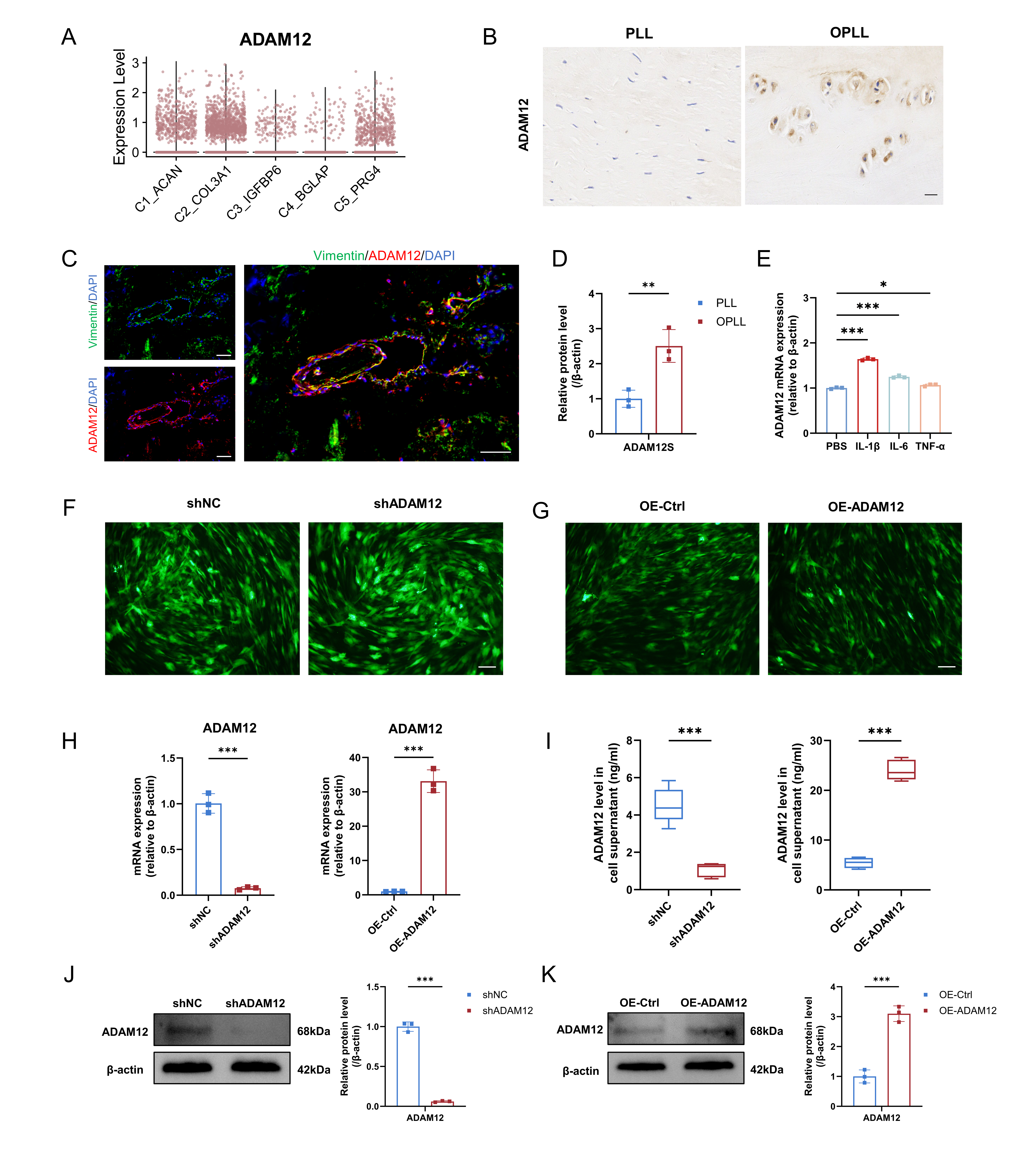

Supplement: Supplementary file 3 — Supplementary Figure 3 [file 41420_2026_3044_MOESM3_ESM.tif]

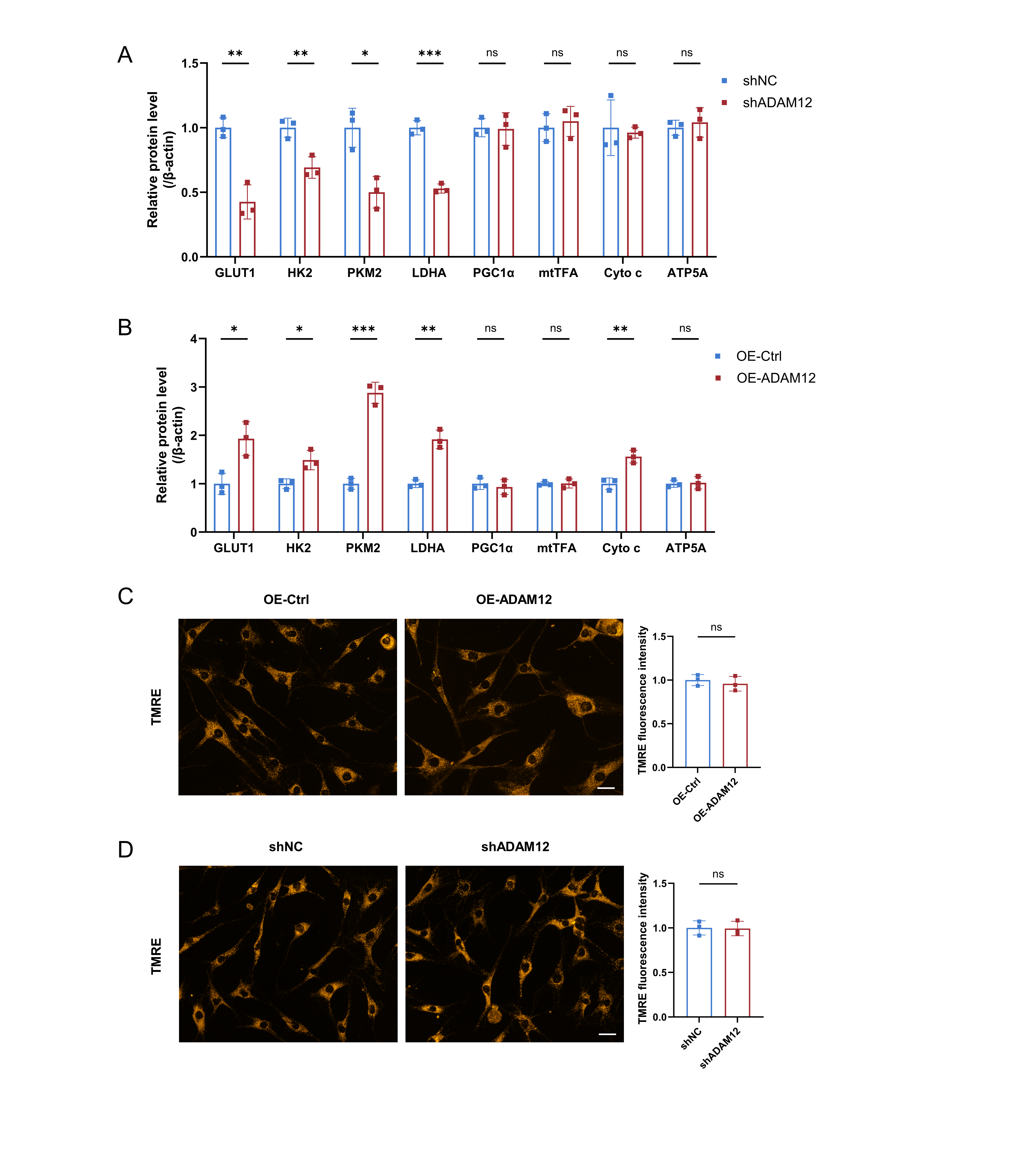

Supplement: Supplementary file 4 — Supplementary Figure 4 [file 41420_2026_3044_MOESM4_ESM.tif]

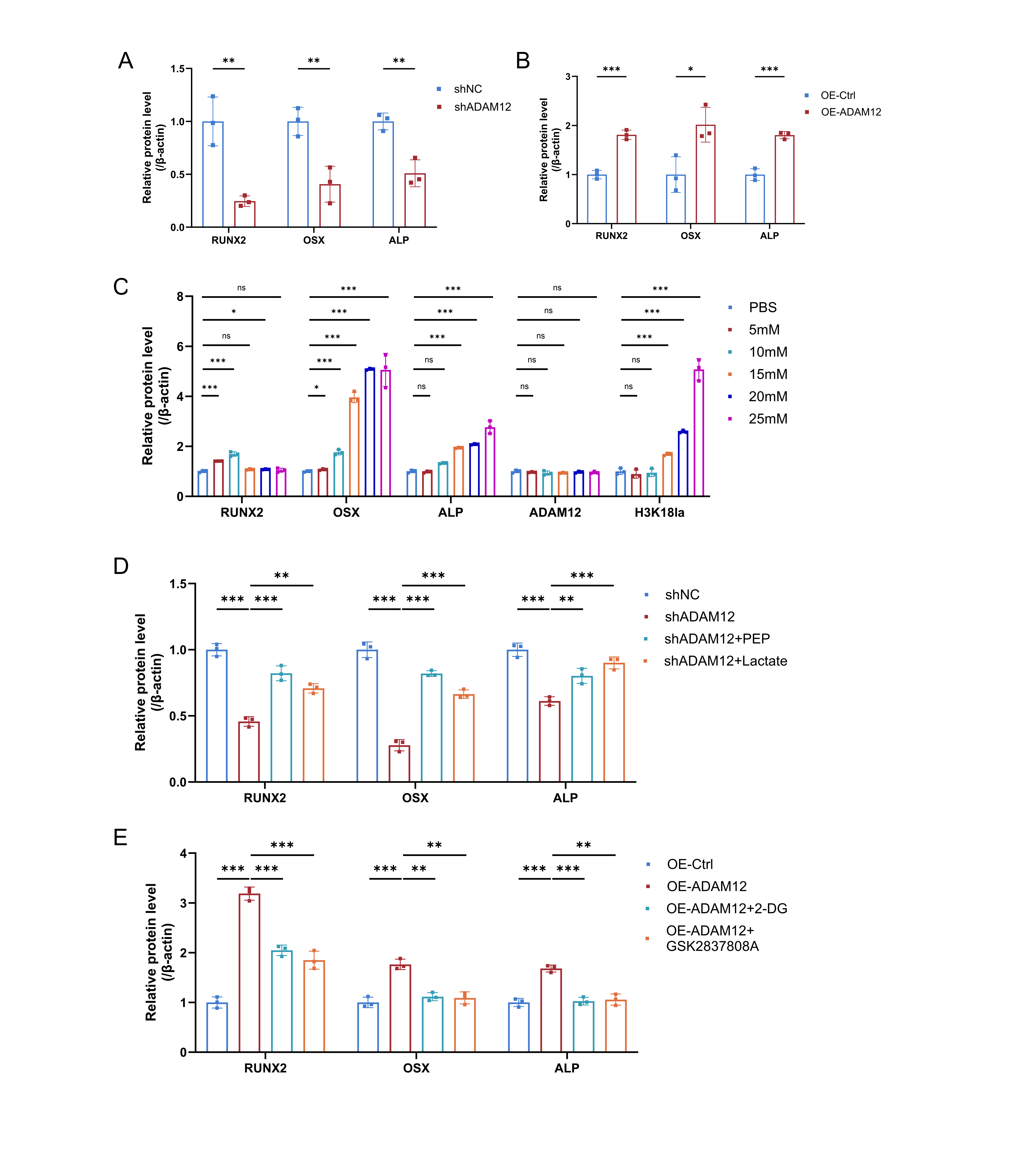

Supplement: Supplementary file 5 — Supplementary Figure 5 [file 41420_2026_3044_MOESM5_ESM.tif]

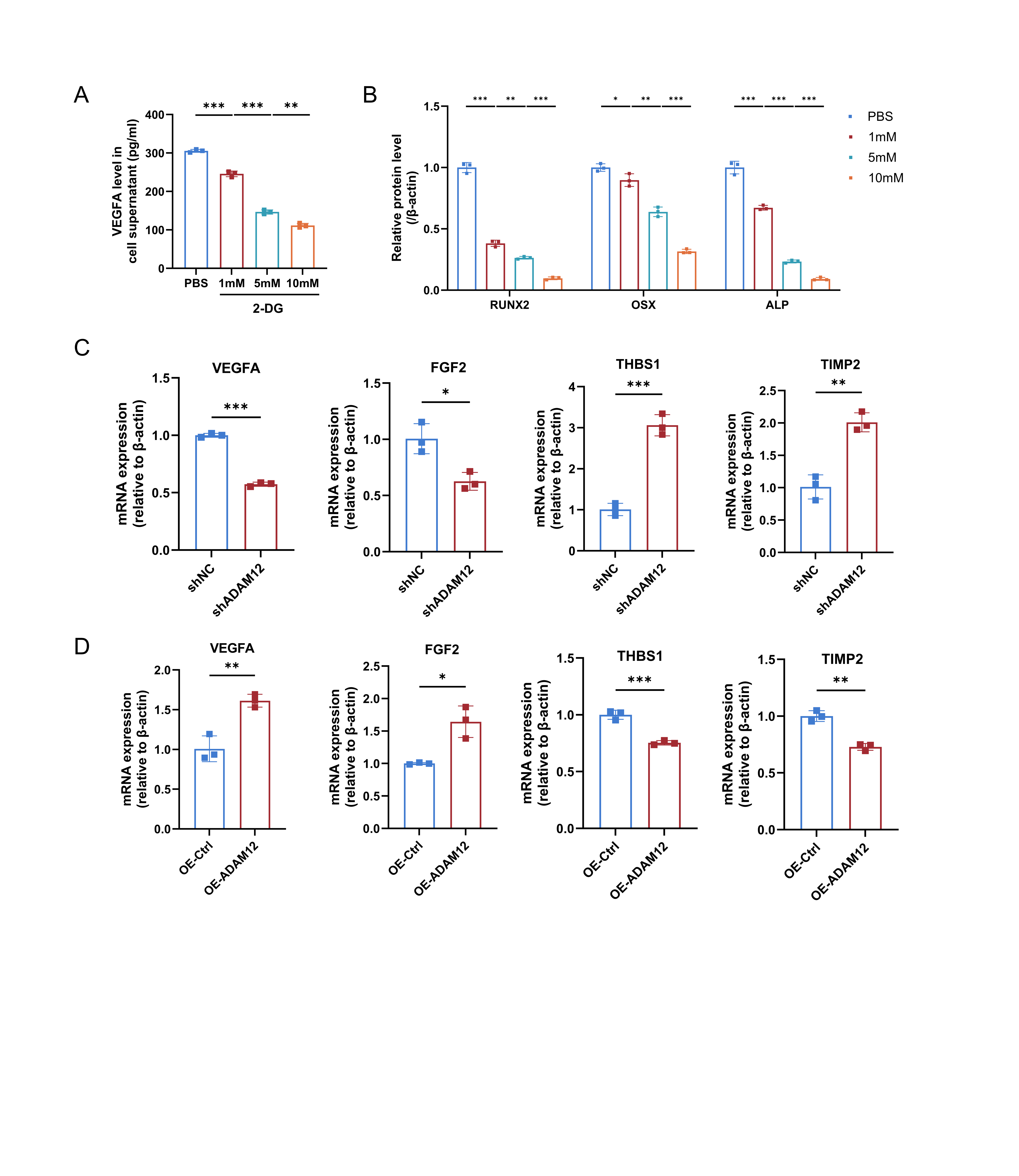

Supplement: Supplementary file 6 — Supplementary Figure 6 [file 41420_2026_3044_MOESM6_ESM.tif]

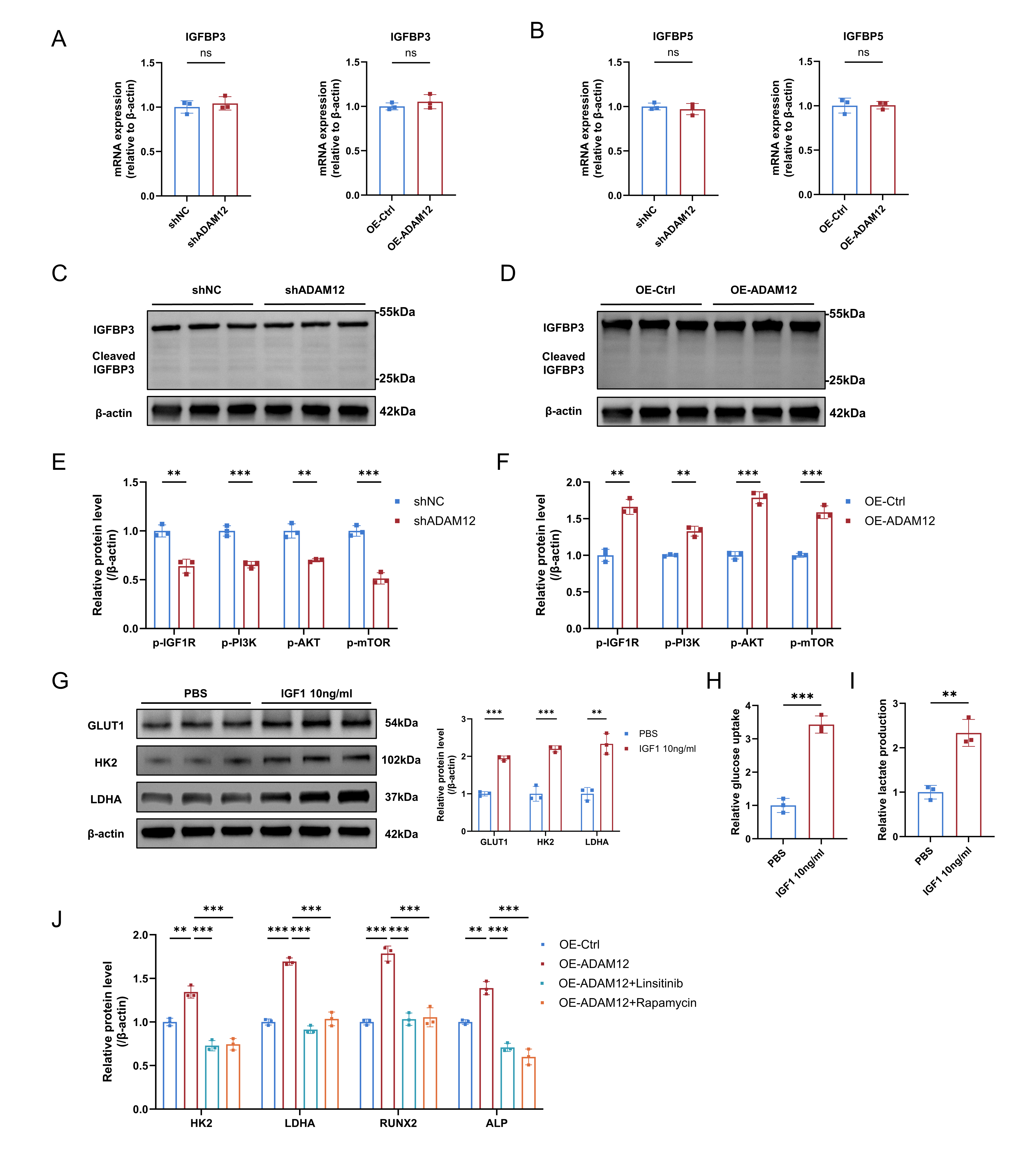

Supplement: Supplementary file 7 — Supplementary Figure 7 [file 41420_2026_3044_MOESM7_ESM.tif]

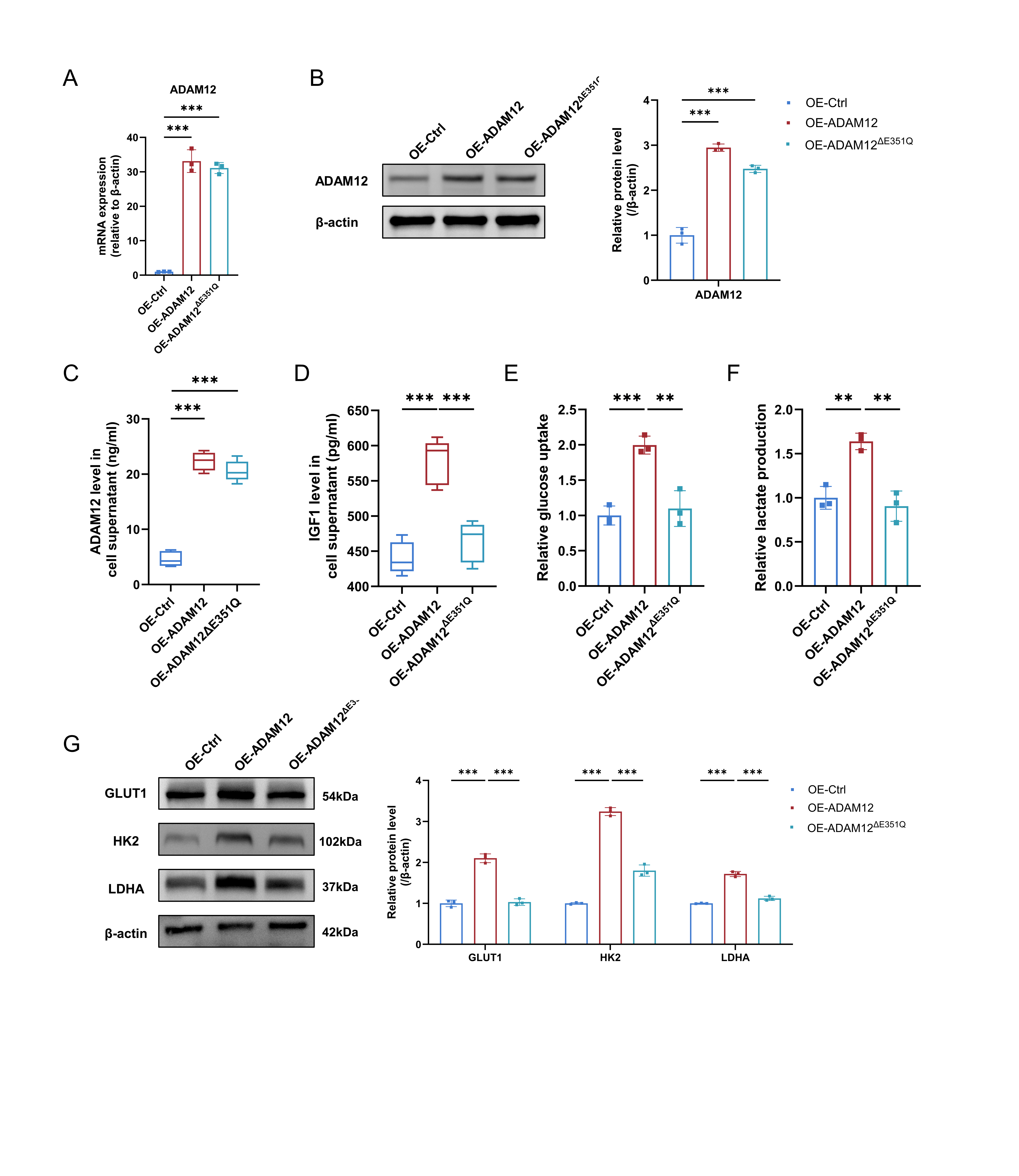

Supplement: Supplementary file 8 — Supplementary Figure 8 [file 41420_2026_3044_MOESM8_ESM.tif]
