# Supplementary material for: Glycolytic reprogramming mediated by the ADAM12/IGF1 axis promotes ossification of the posterior longitudinal ligament
Source: Cell Death Discov. 2026 Mar 25;12:178. doi: 10.1038/s41420-026-03044-8 (PMC13039164; doi:10.1038/s41420-026-03044-8)
Supplement: Supplementary file 9 — Supplementary Figure legend [file 41420_2026_3044_MOESM9_ESM.docx]

Figure S1. Histological and cellular characteristics of OPLL and PLL tissues

A. Bubble plot illustrating the KEGG pathway enrichment analysis of DEGs identified between OPLL and PLL tissues. B. Schematic representation of the cellular subpopulation distribution within the posterior longitudinal ligament tissue, identified by single-cell RNA sequencing. C. Dot plot displaying the expression of canonical marker genes used for annotating the major cell types. D. Representative immunofluorescence images and quantification of RUNX2⁺ osteoprogenitors and CD31⁺ vascular areas in human OPLL and PLL tissues. Scale bar=100 μm, n=3. E. Representative light microscopy images showing the spindle-shaped, fibroblast-like morphology of primary ligament cells. Scale bar=100 μm. Immunofluorescence staining confirms positive expression of the mesenchymal marker Vimentin. Scale bar=50 μm. F. Immunofluorescence analysis confirming the expression of the ligament progenitor marker PRG4 in primary cells. Scale bar=50 μm. G. Representative images of ARS staining and ALP staining confirming the enhanced osteogenic capacity of OPLL cells. Data are presented as mean ± standard deviation.

Figure S2. Quantitative analysis of glucose metabolism and mitochondrial markers during OPLL progression

1. Quantitative analysis of GLUT1, PKM2, and LDHA protein level in OPLL and PLL cells. n=3. B. Quantitative analysis of PDK1, Cyto c, and ATP5A protein level in OPLL and PLL cells. n=3. C. Quantitative analysis of RUNX2, OSX and ALP protein level during a 15day time course of osteogenic differentiation of ligament cells. n=3. D. Quantitative analysis of GLUT1, HK2 and LDHA protein level during a 15day time course of osteogenic differentiation of ligament cells. n=3. E. Quantitative analysis of PDK1, Cyto c and ATP5A protein level during a 15day time course of osteogenic differentiation of ligament cells. n=3. Data are presented as mean ± standard deviation.

Figure S3. Characterization of ADAM12 and validation of stable cell lines.

A. Violin plot displaying the expression distribution of ADAM12 across different ligament cell subpopulations from scRNA-seq data. B. Representative immunohistochemical staining and quantification of ADAM12⁺ cells in human tissue sections. Scale bar=20 μm. C. Immunofluorescence co-localization of ADAM12 with Vimentin. Scale bar=50 μm. D. qPCR analysis of ADAM12 mRNA expression following 24-hour stimulation with inflammatory cytokines (IL-6, IL-1β, TNF-α), suggesting inflammation as a potential inducer. n=3. E. Quantification of ADAM12S protein levels in OPLL and PLL cells. n=3. F, G. Fluorescence microscopy showing transduction efficiency of GFP-tagged lentiviruses (shADAM12/OE-ADAM12) at MOI=40. Scale bar=100 μm. H-K. Validation of ADAM12 knockdown and overexpression efficiency by qPCR (H), ELISA for secreted ADAM12 (I), and Western blot (J, K). n=3. Data are presented as mean ± standard deviation.

Figure S4. ADAM12 modulation alters glycolytic enzymes but not mitochondrial markers

A, B. Quantification of glycolytic (GLUT1, HK2, PKM2, LDHA) and mitochondrial (PGC1α, mtTFA, Cyto c, ATP5A) protein levels in ligament cells following ADAM12 knockdown or overexpression. n=3. C, D. Representative TMRE staining images and quantification of relative fluorescence intensity, showing no significant alteration in mitochondrial membrane potential upon ADAM12 modulation alone. Scale bar=30 μm, n=3. Data are presented as mean ± standard deviation.

Figure S5. ADAM12 promotes the production of angiogenic factors in ligament cells

A. ELISA analysis of VEGFA secretion in the supernatant of ligament cells treated with increasing concentrations of the glycolytic inhibitor 2-DG. n=3. B. Quantitative analysis of RUNX2, OSX and ALP protein level in ligament cells treated with increasing concentrations of the glycolytic inhibitor 2-DG. n=3. C. Quantitative PCR analysis of mRNA expression levels for the pro-angiogenic factors VEGFA and FGF2, and the anti-angiogenic factors THBS1 and TIMP2, in ligament cells following ADAM12 knockdown under osteogenic conditions. n=3. D. Quantitative PCR analysis of mRNA expression levels for VEGFA, FGF2, THBS1, and TIMP2 in ligament cells following ADAM12 overexpression under osteogenic conditions. n=3. Data are presented as mean ± standard deviation.

Figure S6. ADAM12 promotes osteogenic differentiation of ligament cells via glycolysis-mediated lactate production

A, B. Quantitative analysis of RUNX2, OSX and ALP protein level in ligament cells after ADAM12 knockdown and overexpression. n=3. C. Quantitative analysis of RUNX2, OSX, ALP, ADAM12 and H3K18la protein level in ligament cells treated with increasing concentrations of lactate. D. Quantitative analysis of RUNX2, OSX and ALP protein level in ADAM12-knockdown cells after treatment with PEP or lactate. n=3. E. Quantitative analysis of RUNX2, OSX and ALP protein level in ADAM12-overexpressing cells treated with 2-DG or GSK2837808A. n=3. Data are presented as mean ± standard deviation.

Figure S7. ADAM12 regulates glycolysis in ligament cells via the IGF1 signaling pathway

A-C. Validation of IGF1 effects: Western blot of glycolytic enzymes (A), glucose uptake (B), and lactate production (C) in cells treated with recombinant IGF1. n=3. D, E. qPCR analysis showing no significant change in IGFBP3 mRNA levels upon ADAM12 modulation. F, G. qPCR analysis of IGFBP5 mRNA levels. H, I. Western blot analysis confirming that ADAM12 modulation does not affect IGFBP3 protein cleavage or levels. J, K. Quantification of phosphorylation levels of IGF1R/PI3K/AKT/mTOR signaling nodes. L. Quantification of HK2, LDHA, RUNX2, and ALP proteins in ADAM12-overexpressing cells treated with Linsitinib or Rapamycin. Data are presented as mean ± standard deviation.

Figure S8. ADAM12-mediated metabolic reprogramming depend on its metalloproteinase activity

A, B. Validation of the catalytically inactive mutant (OE-ADAM12^ΔE351Q^) expression at transcriptional and protein levels. n=3. C, D. ELISA analysis of secreted ADAM12 (C) and IGF1 (D) levels in supernatants from OE-ADAM12 and OE-ADAM12^ΔE351Q^ cells. n=5. E-G. Analysis of glucose uptake (E), lactate production (F), and glycolytic enzyme levels (G) showing that the catalytic mutant fails to drive glycolytic flux. n=3. Data are presented as mean ± standard deviation.
