# Supplementary material for: Glycolytic reprogramming mediated by the ADAM12/IGF1 axis promotes ossification of the posterior longitudinal ligament
Source: Cell Death Discov. 2026 Mar 25;12:178. doi: 10.1038/s41420-026-03044-8 (PMC13039164; doi:10.1038/s41420-026-03044-8)
Supplement: Supplementary file 10 — Supplementary Table [file 41420_2026_3044_MOESM10_ESM.docx]

Table S1. Baseline characteristics of the patients for the study

| Characteristic | OPLL patients  (n=10) | non-OPLL patients  (n=10) | p Value |
| --- | --- | --- | --- |
| Age (years) | 54.3±6.3 | 52.4±7.0 | 0.53 |
| male:female | 6:4 | 5:5 | 1.00 |
| Body-mass index | 22.5±2.0 | 22.2±1.8 | 0.78 |
| Smoker (n) | 3 | 5 | 0.65 |
| Alcoholism (n) | 3 | 2 | 1.00 |
| Hypertension (n) | 2 | 4 | 0.10 |
| Diabetes (n) | 4 | 3 | 0.63 |
| JOA score | 11.2±2.3 | 12.1±2.1 | 0.37 |

Table S2. Human real-time PCR primer sequences

| Name | Forward primer | Reverse primer |
| --- | --- | --- |
| β-actin | 5′-GAGACCTTCAACACCCCAGC-3′ | 5′-GGAGAGCATAGCCCTCGTAGAT-3′ |
| ADAM12-L | 5′-CCTTATGGCAACTGTGGCAAAGTC-3′ | 5′-GGCTGGCACCTCCTTGACAC-3′ |
| ADAM12-S | 5′-CAAGCAGAAGCAAGGCAGGAAG-3′ | 5′-AGATGAGTGTCAGTGAGGCAGTAG-3′ |
| RUNX2 | 5′-TCAACGATCTGAGATTTGTGGG-3′ | 5′-GGGGAGGATTTGTGAAGACGG-3′ |
| OSX | 5′-CGGCAAGAGGTTCACTCGTTCG-3′ | 5′-TGGAGCAGAGCAGGCAGGTG-3′ |
| ALP | 5′-TGGTGGAAGGAGGCAGA-3′ | 5′-GTGAAGACGTGGGAATGGT-3′ |
| VEGFA | 5′-ACAAATGTGAATGCAGACCAAA-3′ | 5′-ACCAACGTACACGCTCCAG-3′ |
| FGF2 | 5′-GAGGACCCATAAGAGTTCACA-3′ | 5′-TGCAGCCTTACCCAATCTA-3′ |
| THBS1 | 5′-TCCCTACAACCACAACCC-3′ | 5′-TCACACTGATCTCCAACCC-3′ |
| TIMP2 | 5′-AGCTCTGACATCCCTTCCT-3′ | 5′-GTCTCCCTCCAGACCCA-3′ |
| IGFBP3 | 5'-CAAGCGGGAGACAGAATATGG-3' | 5'-GGACTTATTTCTTAATCCCTC CCT-3' |
| IGFBP5 | 5'-CAAGAGAAAGCCTCTCTCCAG-3' | 5'-CACATTTGAGAAGCCCCTCC-3' |
