# Supplementary material for: Glycolytic reprogramming mediated by the ADAM12/IGF1 axis promotes ossification of the posterior longitudinal ligament
Source: Cell Death Discov. 2026 Mar 25;12:178. doi: 10.1038/s41420-026-03044-8 (PMC13039164; doi:10.1038/s41420-026-03044-8)

Figure 2B

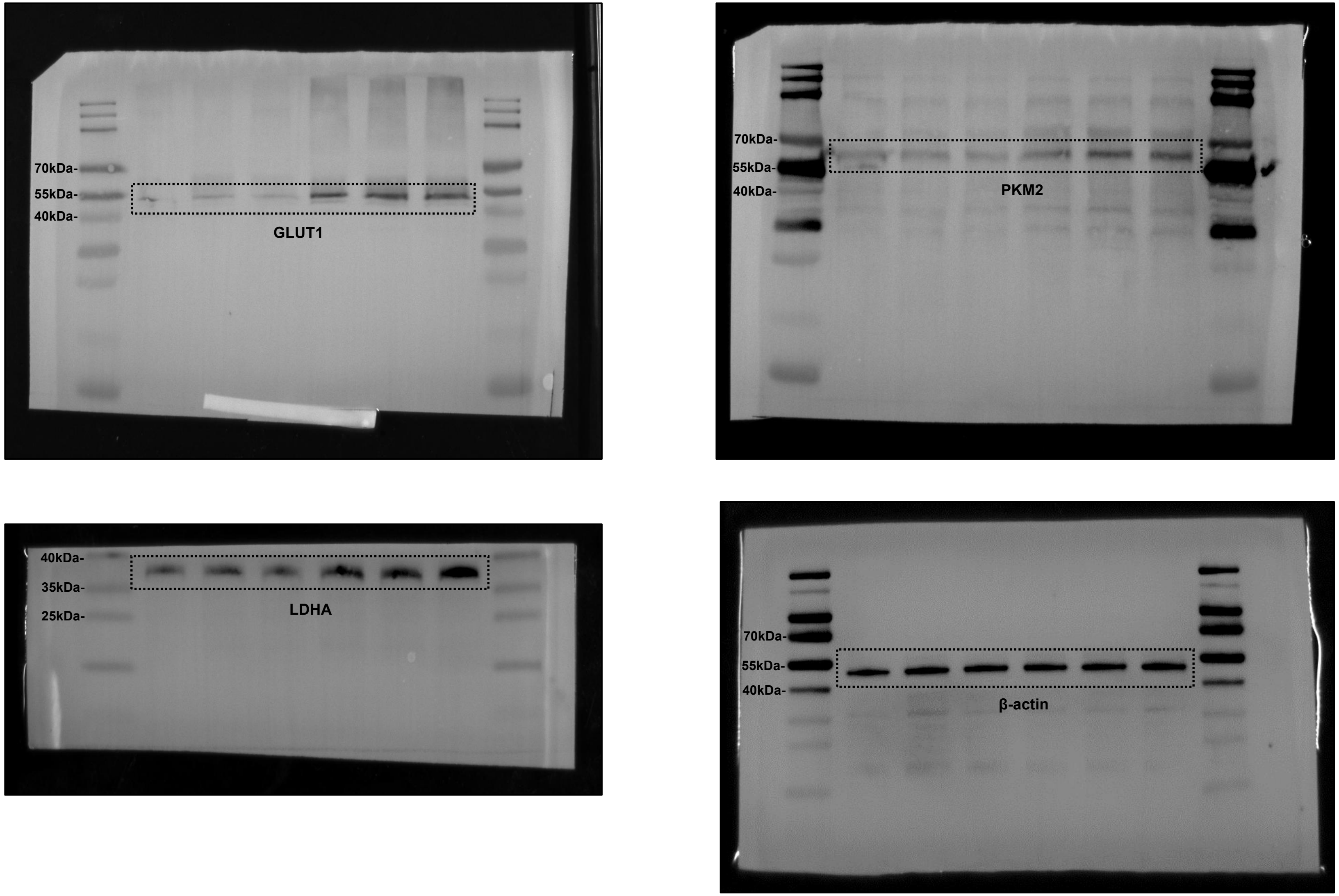
70kDa-
55kDa-
40kDa-
GLUT1
70kDa-
55kDa-
40kDa-
PKM2
40kDa-
35kDa-
25kDa-
LDHA
70kDa-
55kDa-
40kDa-
β-actin

Figure 2G

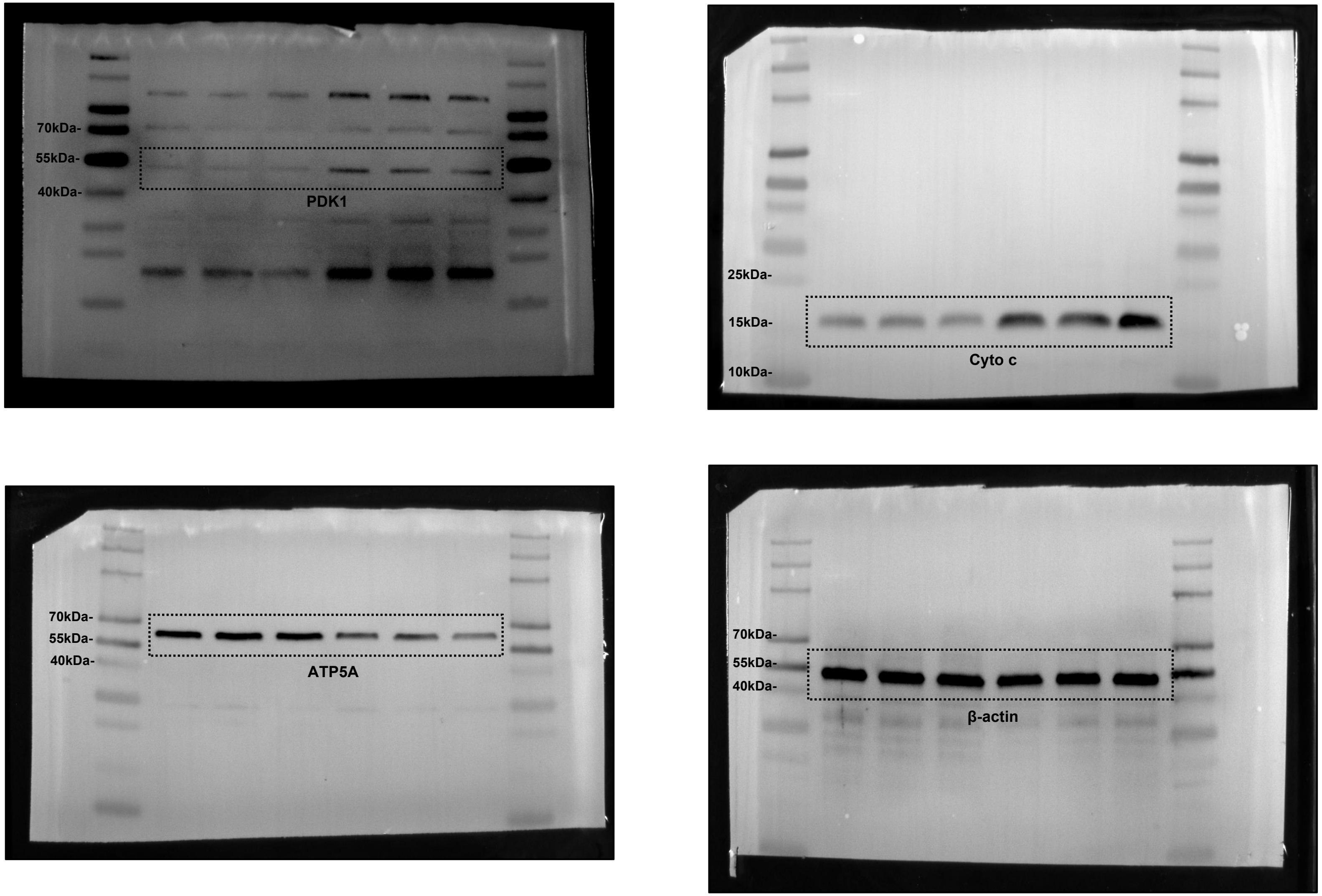
70kDa-
55kDa-
40kDa-
PDK1
25kDa-
15kDa-
10kDa-
Cyto c
70kDa-
55kDa-
40kDa-
ATP5A
70kDa-
55kDa-
40kDa-
β-actin

Figure 2J

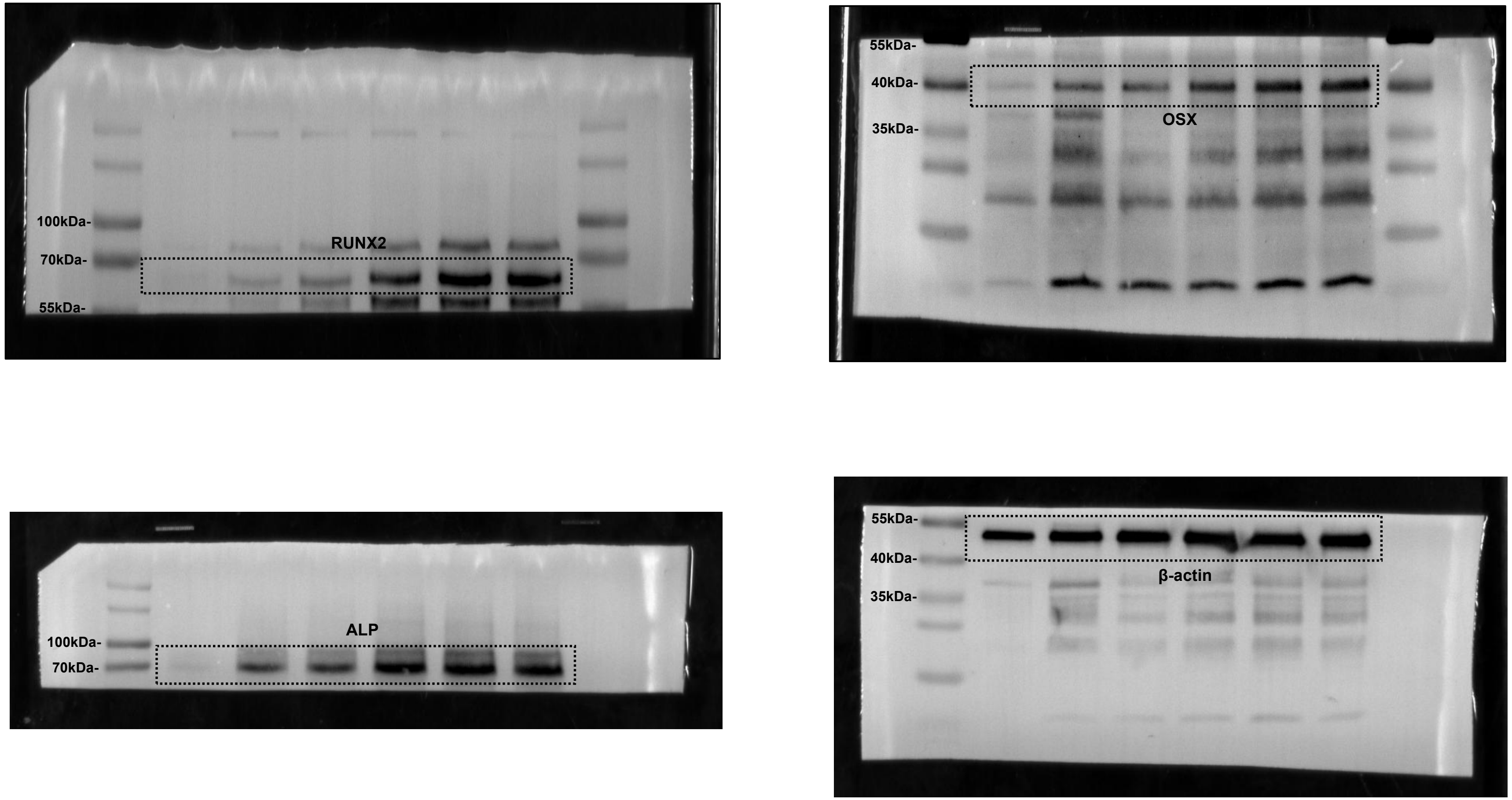
100kDa-
70kDa-
55kDa-
RUNX2
55kDa-
40kDa-
35kDa-
OSX
100kDa-
70kDa-
ALP
55kDa-
40kDa-
35kDa-
β-actin

Figure 2K

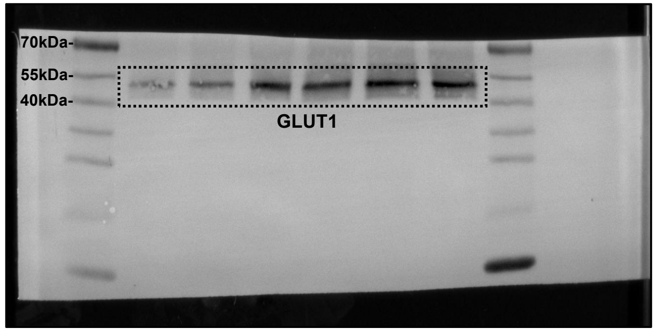

70kDa-
55kDa-
40kDa-
GLUT1

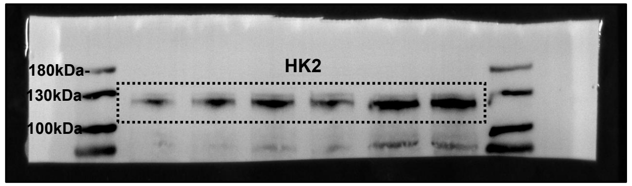

HK2
180kDa-
130kDa-
100kDa-

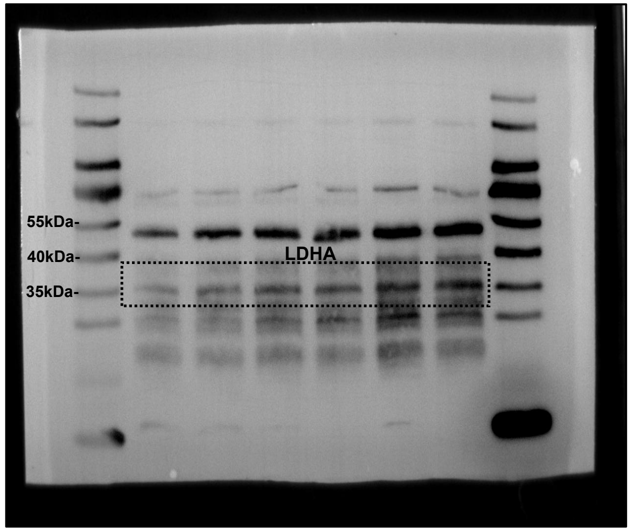

55kDa-
40kDa-
35kDa-
LDHA

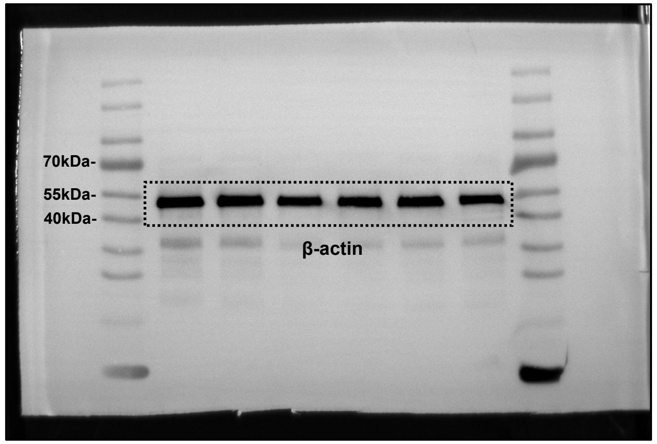

70kDa-
55kDa-
40kDa-
β-actin

Figure 2L

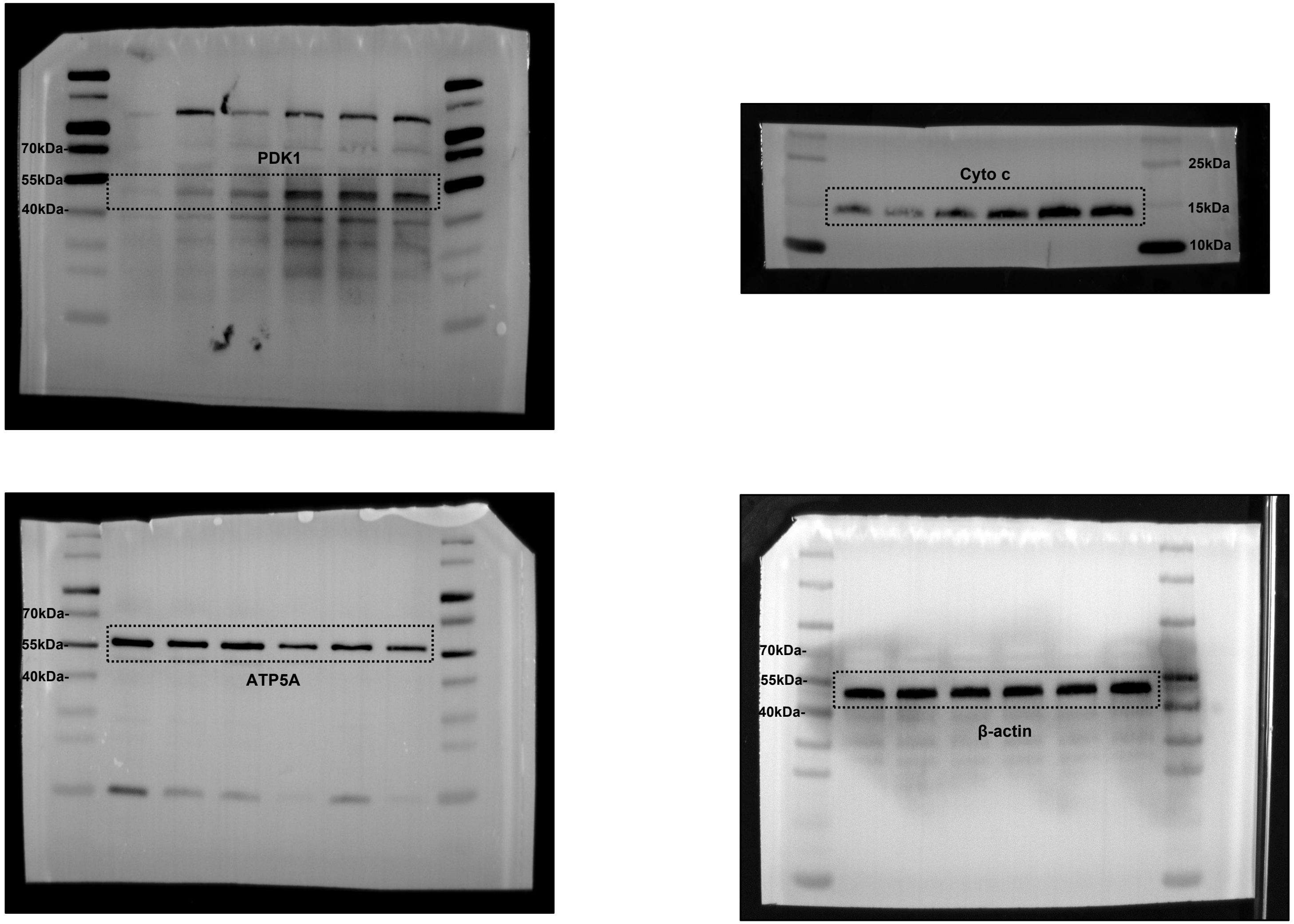

PDK1
70kDa-
55kDa-
40kDa-
Cyto c
25kDa
15kDa
10kDa
70kDa-
55kDa-
40kDa-
ATP5A
70kDa-
55kDa-
40kDa-
β-actin

Figure 3A

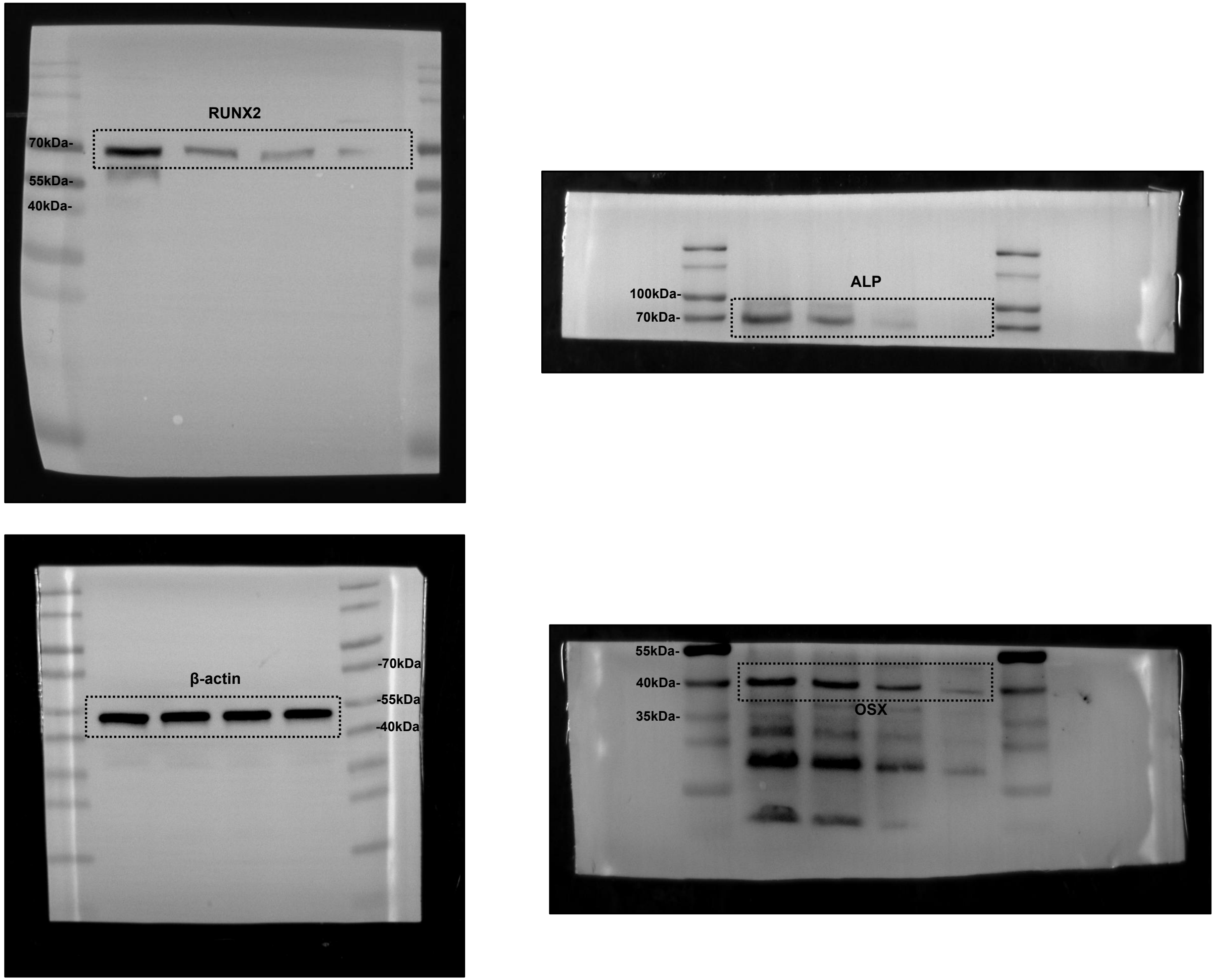
RUNX2
70kDa-
55kDa-
40kDa-
ALP
100kDa-
70kDa-
β-actin
-70kDa
-55kDa
-40kDa
55kDa-
40kDa-
35kDa-
OSX

Figure 4J

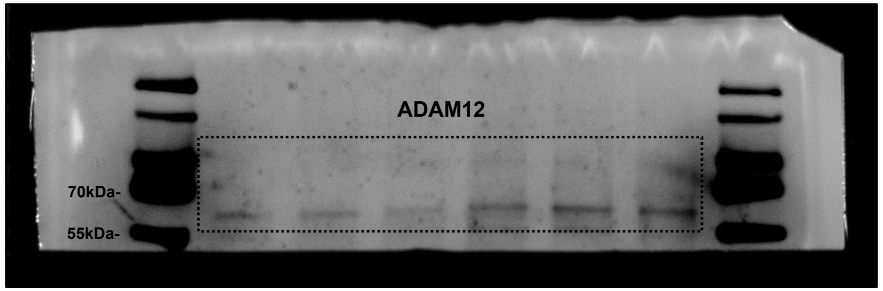

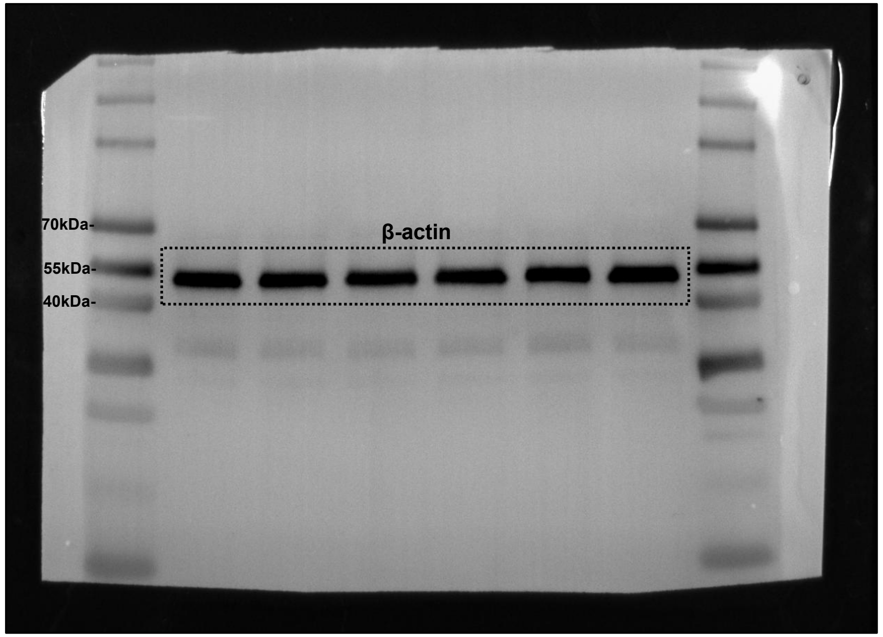

Figure 5C

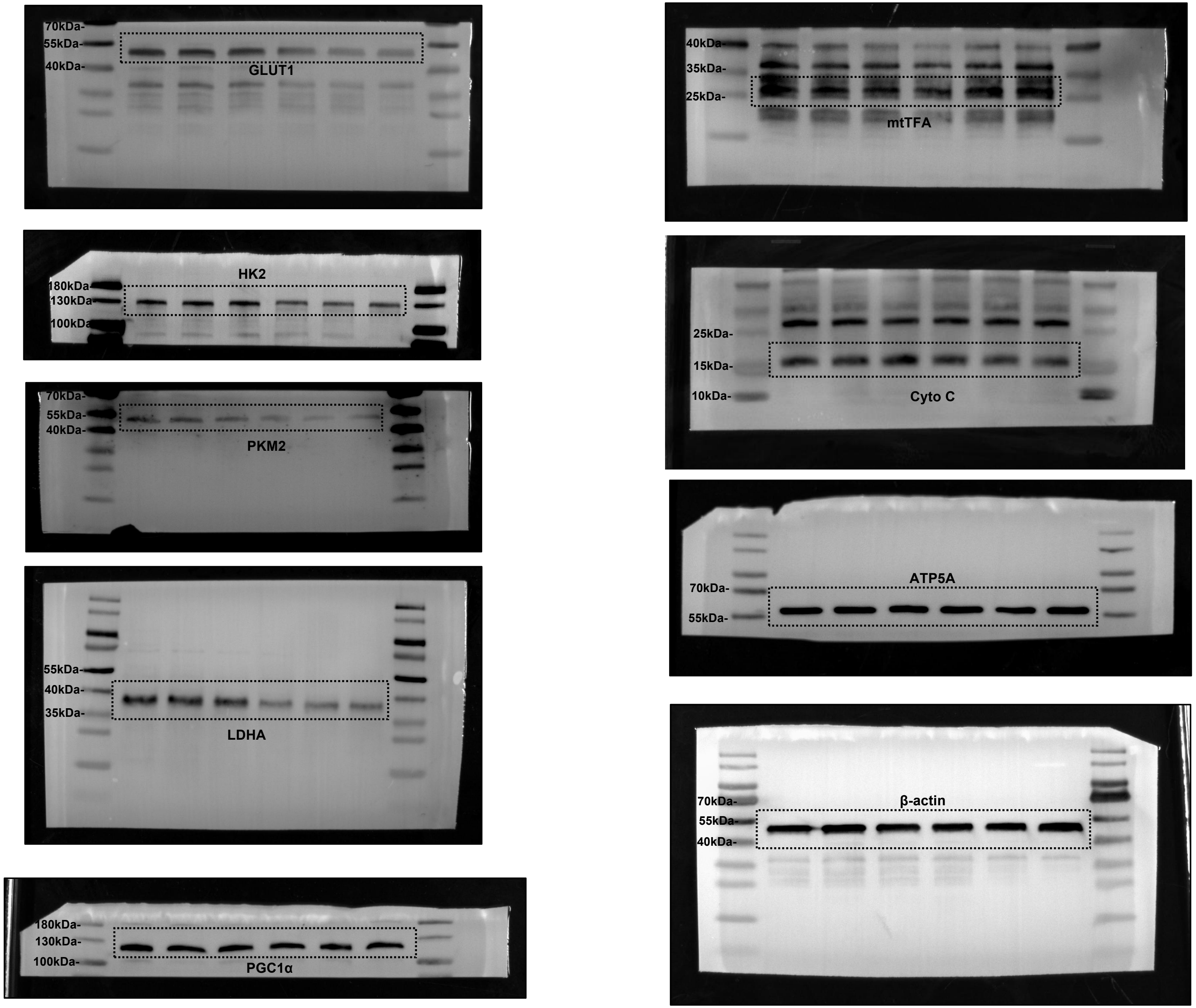
70kDa-
55kDa-
40kDa-
GLUT1
40kDa-
35kDa-
25kDa-
mtTFA
HK2
180kDa-
130kDa-
100kDa-
25kDa-
15kDa-
10kDa-
Cyto C
70kDa-
55kDa-
40kDa-
PKM2
ATP5A
70kDa-
55kDa-
55kDa-
40kDa-
35kDa-
LDHA
70kDa-
β-actin
55kDa-
40kDa-
180kDa-
130kDa-
100kDa-
PGC1α

Figure 5D

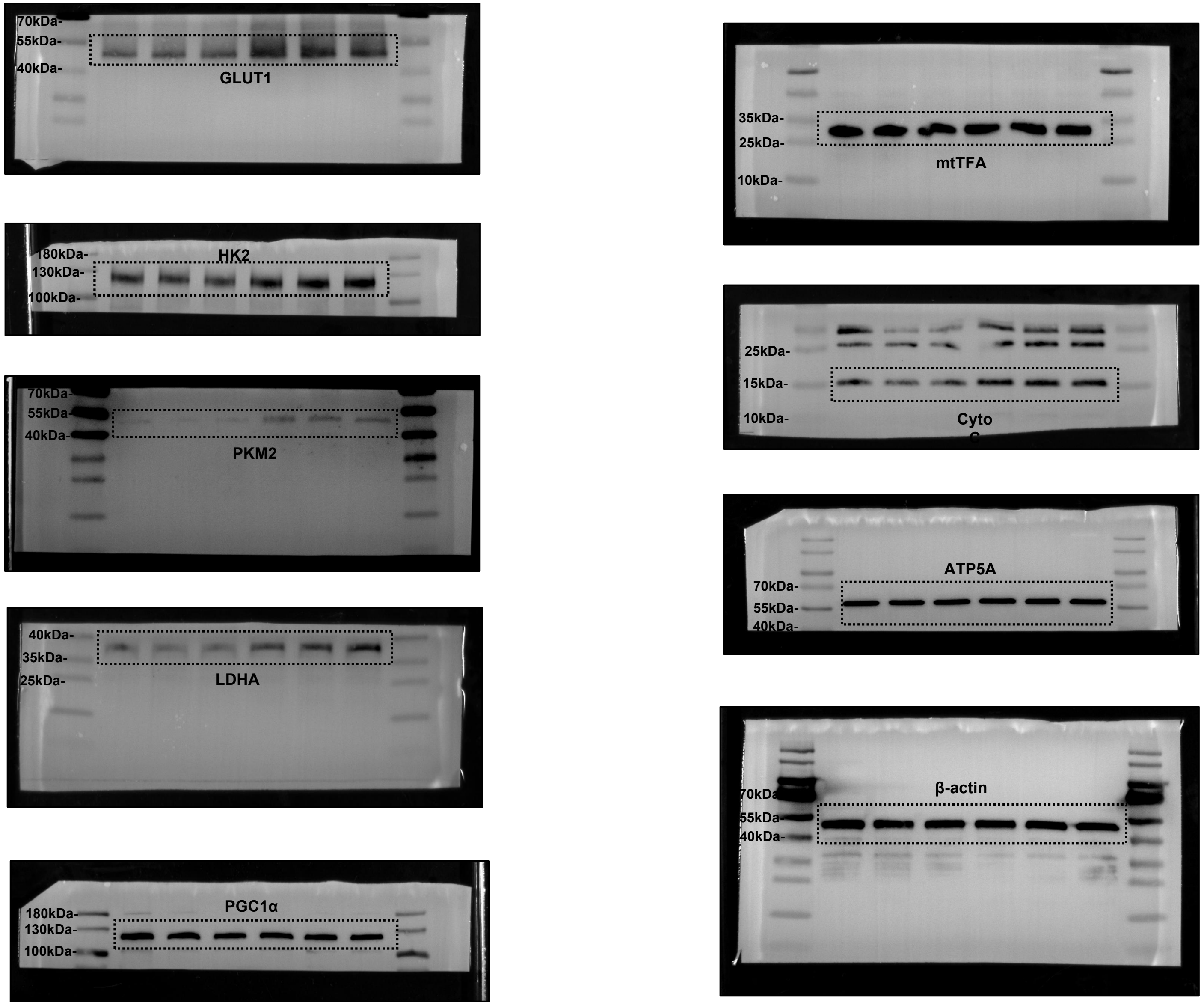
70kDa-
55kDa-
40kDa-
GLUT1
180kDa-
130kDa-
100kDa-
HK2
70kDa-
55kDa-
40kDa-
PKM2
40kDa-
35kDa-
25kDa-
LDHA
180kDa-
130kDa-
100kDa-
PGC1α
35kDa-
25kDa-
10kDa-
mtTFA
25kDa-
15kDa-
10kDa-
Cyto
70kDa-
55kDa-
40kDa-
ATP5A
70kDa-
55kDa-
40kDa-
β-actin

Figure 6B

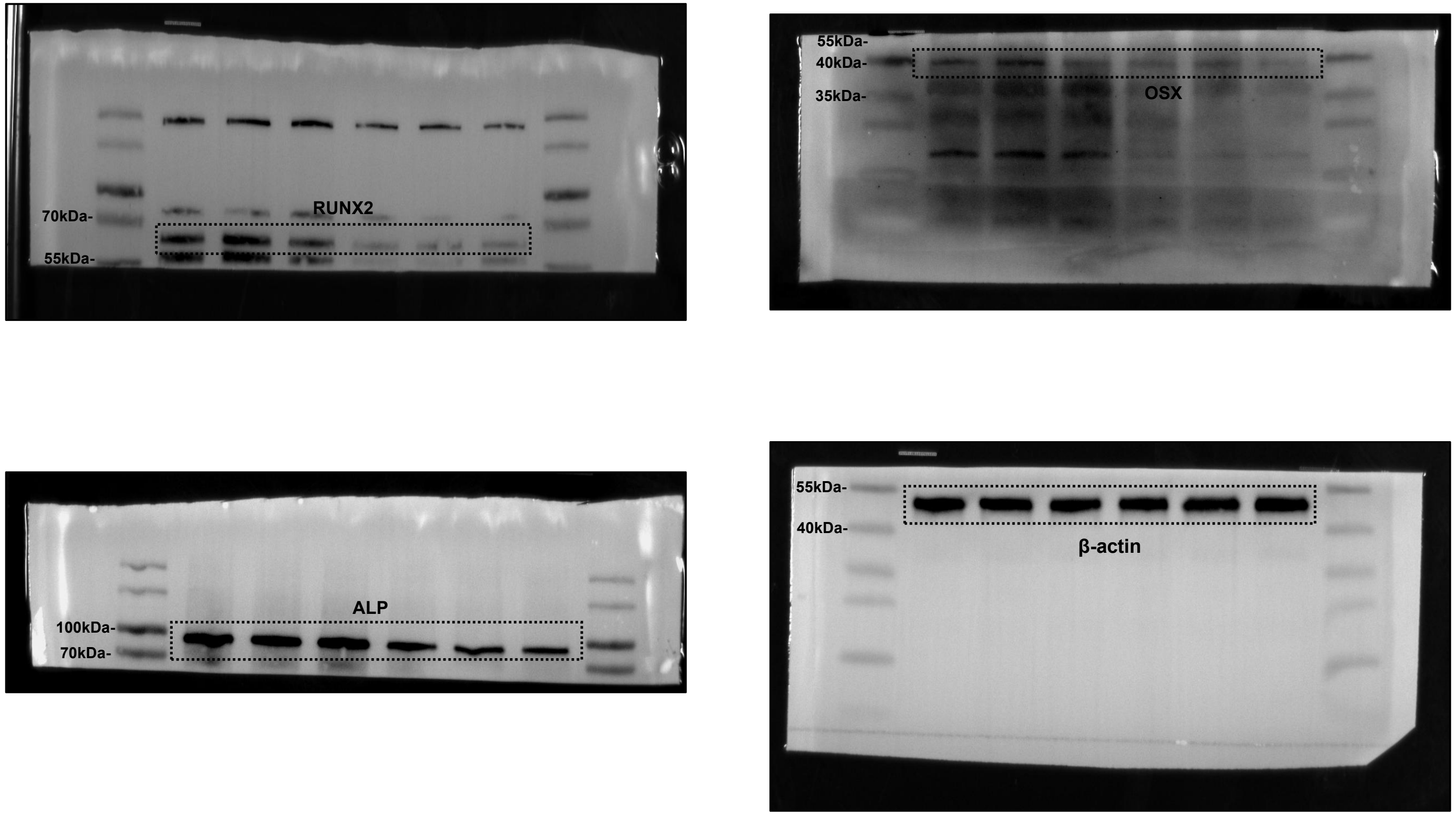
70kDa-
55kDa-
RUNX2
55kDa-
40kDa-
35kDa-
OSX
100kDa-
70kDa-
ALP
55kDa-
40kDa-
β-actin

Figure 6E

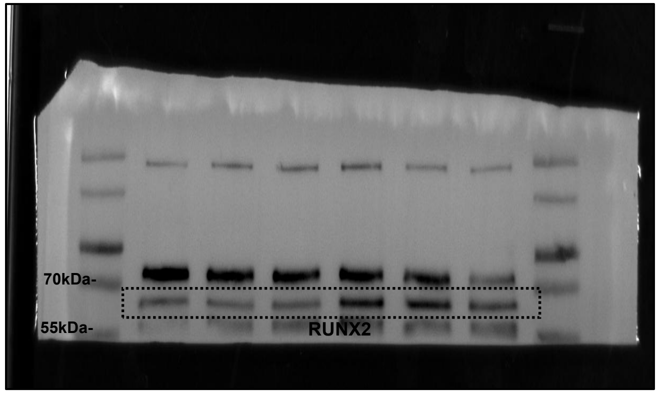
70kDa-
55kDa-
RUNX2

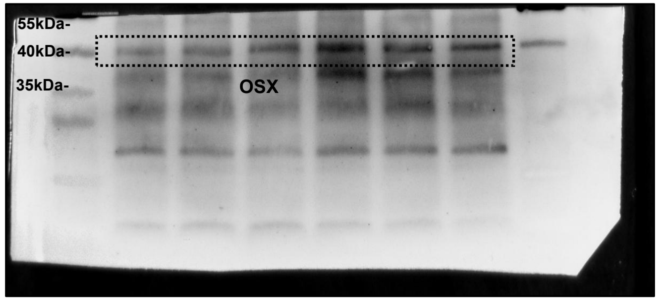
55kDa-
40kDa-
35kDa-
OSX

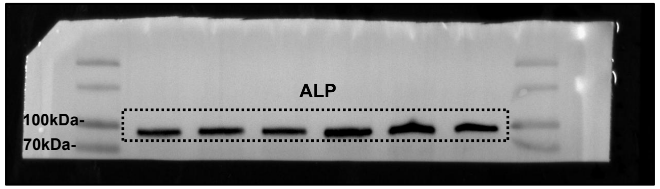
ALP
100kDa-
70kDa-

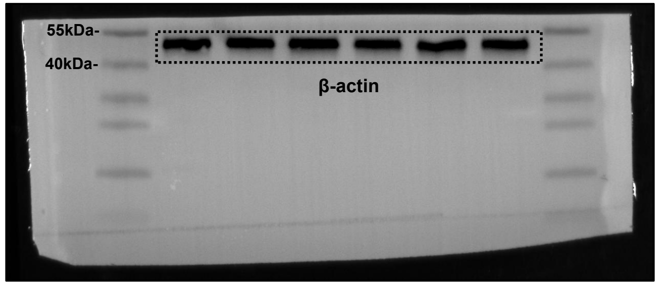
55kDa-
40kDa-
β-actin

Figure 6G

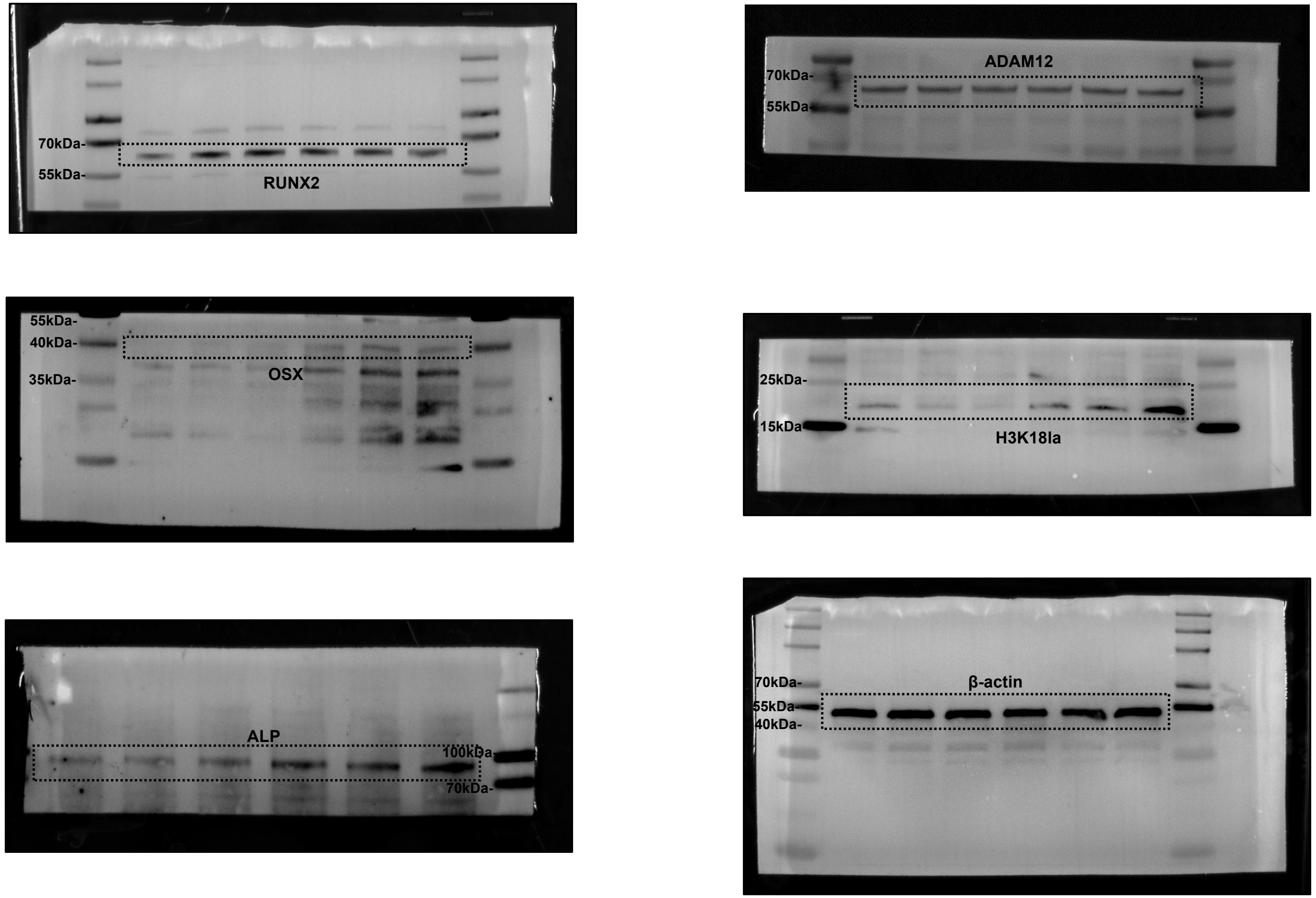
70kDa-
55kDa-
RUNX2
ADAM12
70kDa-
55kDa-
55kDa-
40kDa-
35kDa-
OSX
25kDa-
15kDa
H3K18la
ALP
100kDa-
70kDa-
70kDa-
β-actin
55kDa-
40kDa-

Figure 6H

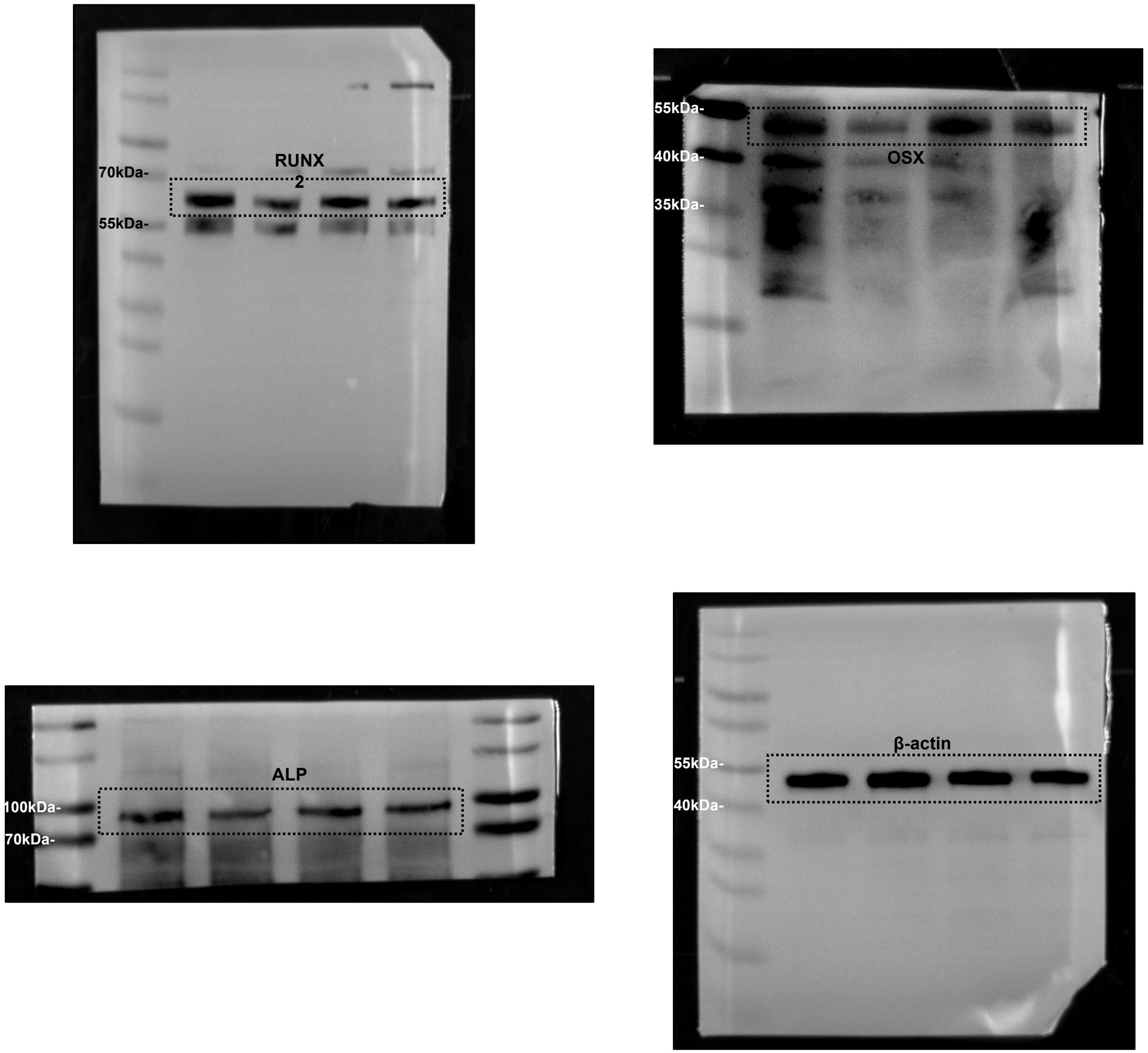
RUNX
2
70kDa-
55kDa-
55kDa-
40kDa-
35kDa-
OSX
ALP
100kDa-
70kDa-
β-actin
55kDa-
40kDa-

Figure 6I

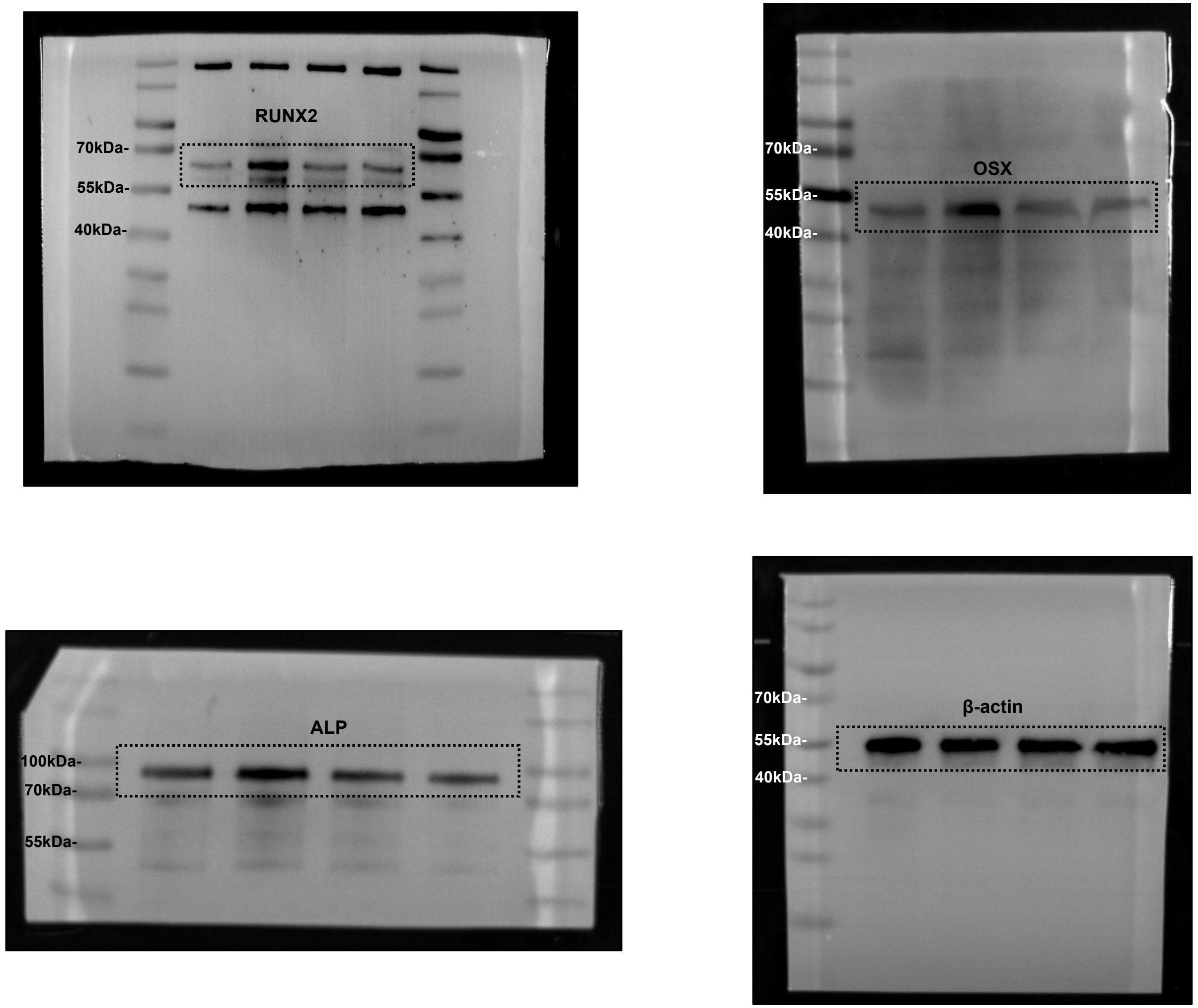
RUNX2
70kDa-
55kDa-
40kDa-
OSX
70kDa-
55kDa-
40kDa-
ALP
100kDa-
70kDa-
55kDa-
β-actin
70kDa-
55kDa-
40kDa-

Figure 7D

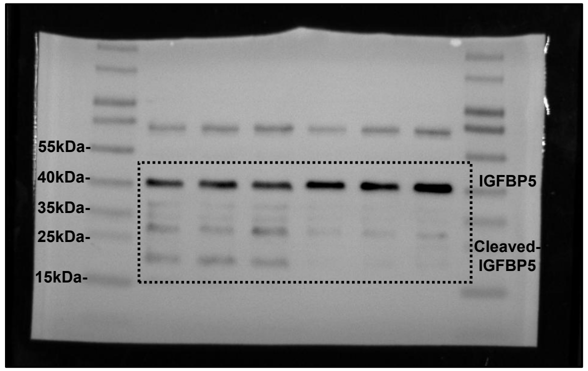

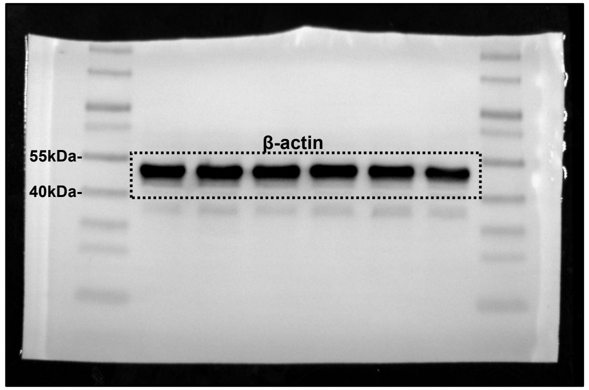

Figure 7E

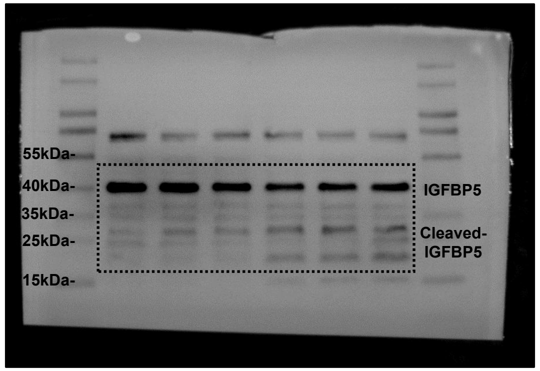

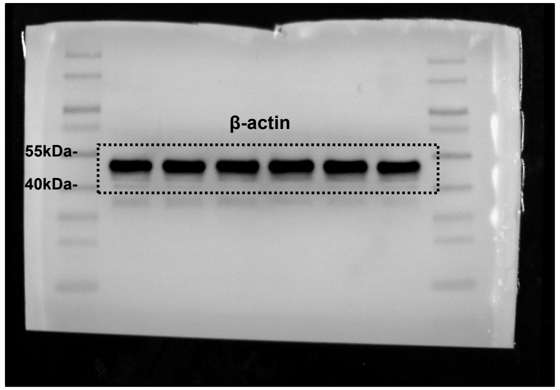

Figure 7F

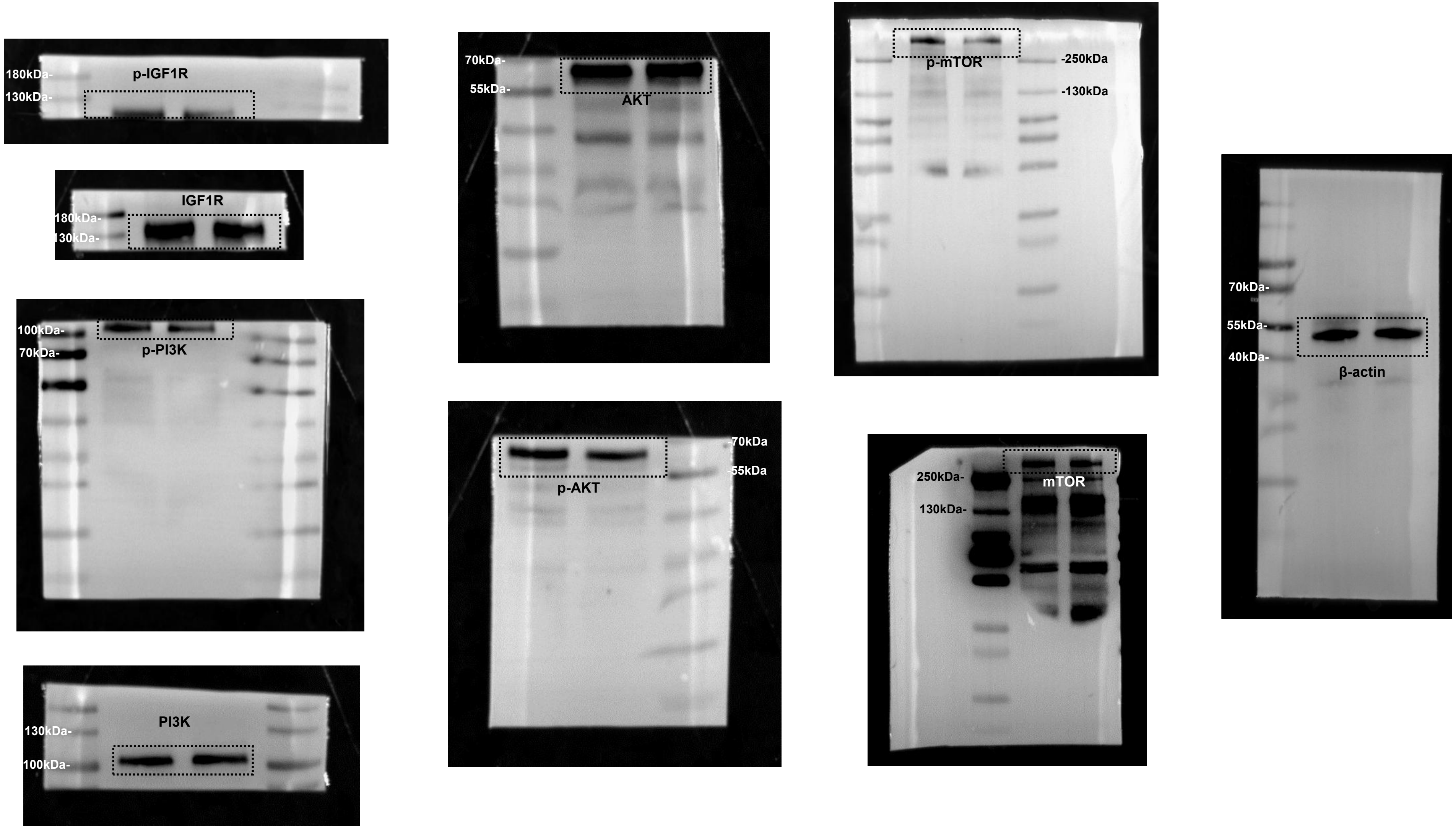

p-IGF1R
180kDa-
130kDa-
IGF1R
180kDa-
130kDa-
100kDa-
70kDa-
p-PI3K
PI3K
130kDa-
100kDa-
70kDa-
55kDa-
AKT
-70kDa
-55kDa
p-AKT
-250kDa
-130kDa
p-mTOR
250kDa-
130kDa-
mTOR
70kDa-
55kDa-
40kDa-
β-actin

Figure 7G

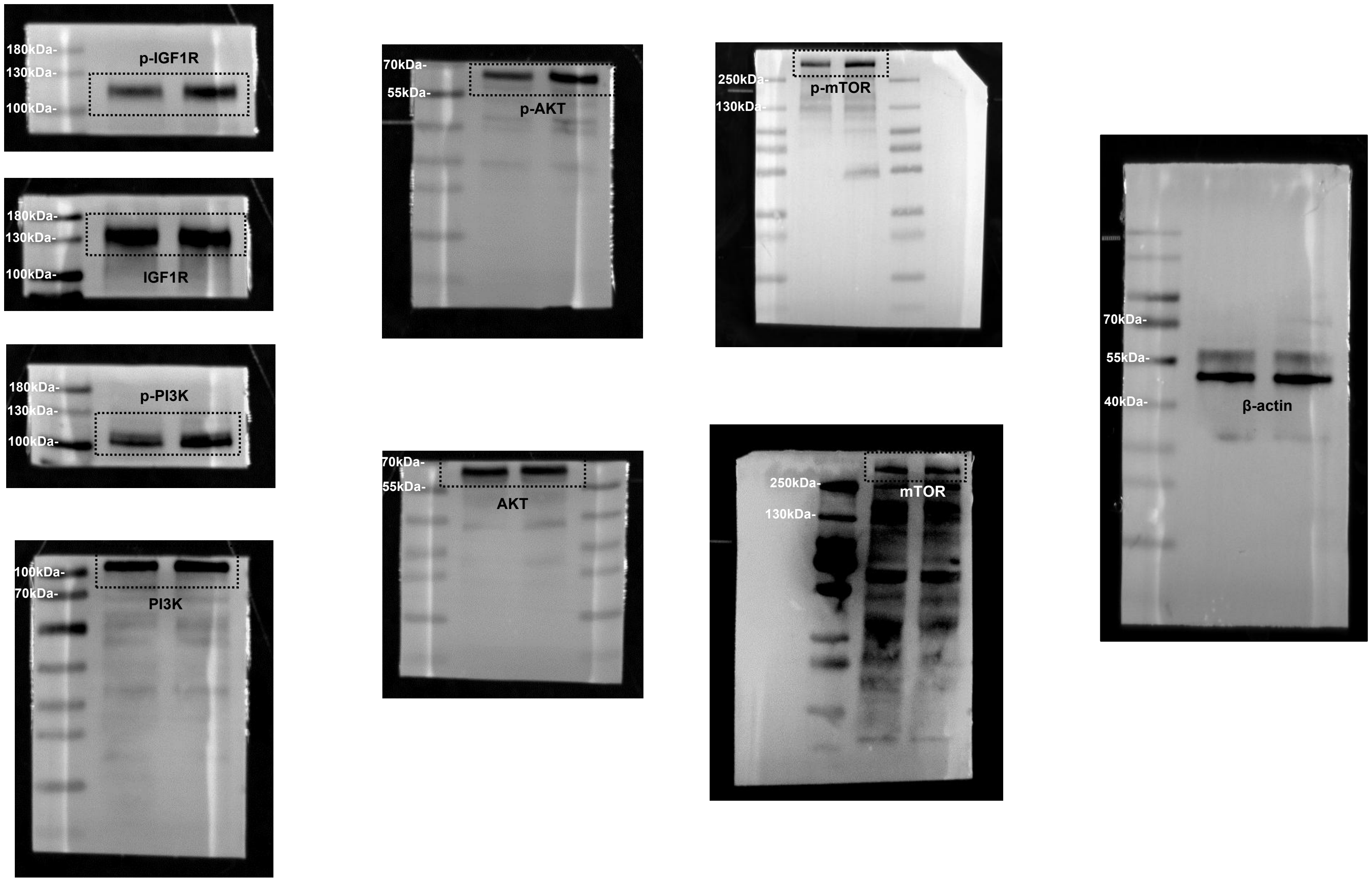
p-IGF1R
180kDa-
130kDa-
100kDa-
IGF1R
180kDa-
130kDa-
100kDa-
p-PI3K
180kDa-
130kDa-
100kDa-
PI3K
100kDa-
70kDa-
p-AKT
70kDa-
55kDa-
AKT
70kDa-
55kDa-
p-mTOR
250kDa-
130kDa-
mTOR
250kDa-
130kDa-
β-actin
70kDa-
55kDa-
40kDa-

Figure 7J

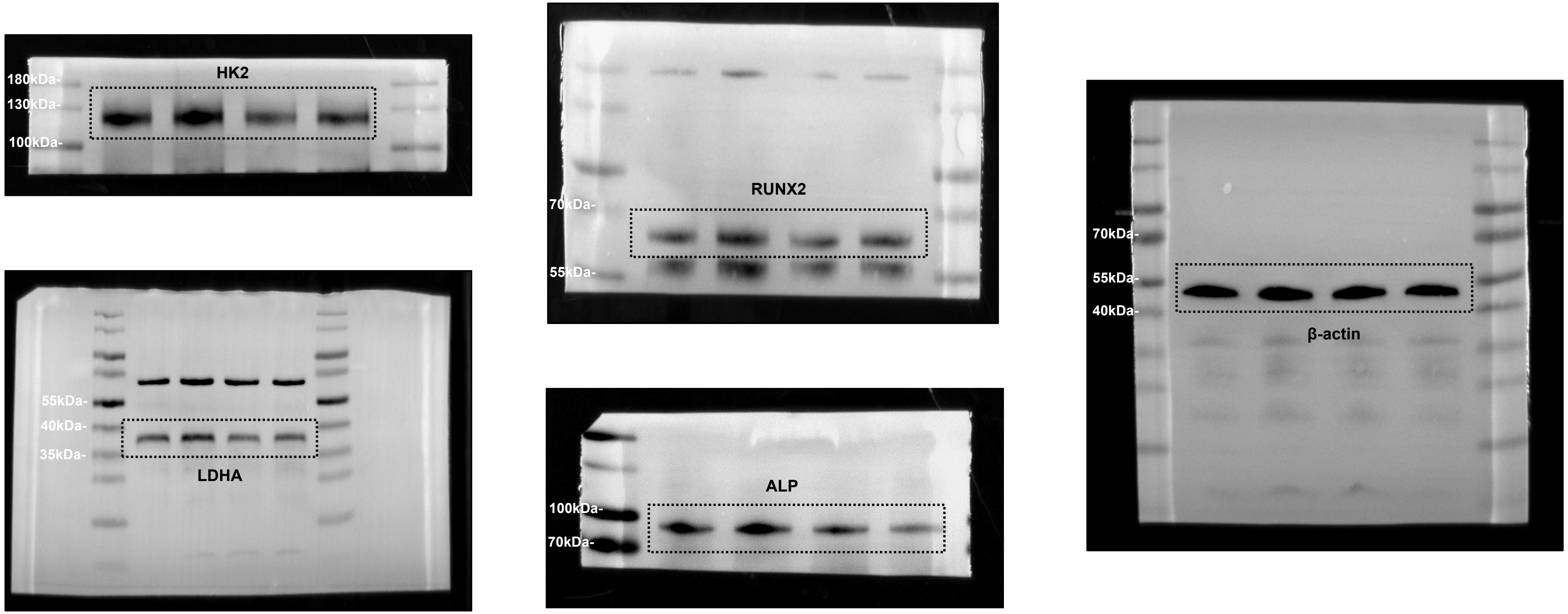

HK2
180kDa-
130kDa-
100kDa-
LDHA
55kDa-
40kDa-
35kDa-
RUNX2
70kDa-
55kDa-
ALP
100kDa-
70kDa-
β-actin
70kDa-
55kDa-
40kDa-

Figure S3J

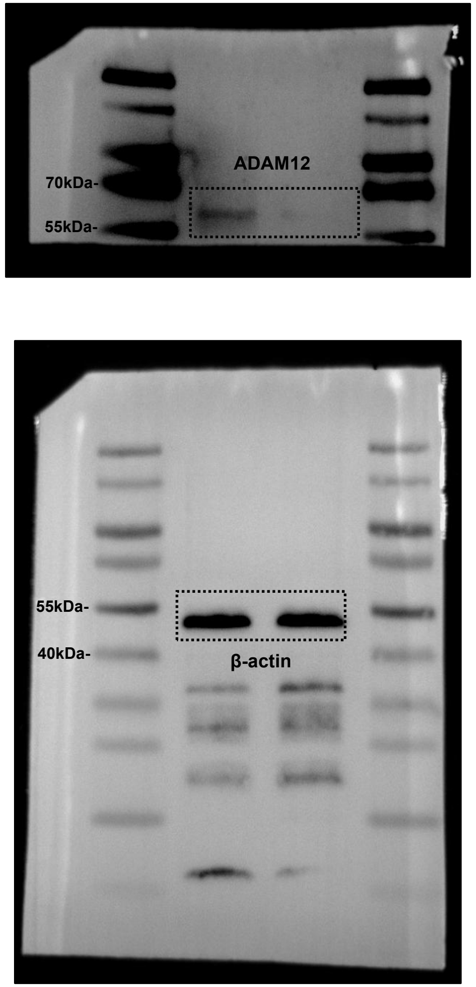

Figure S3K

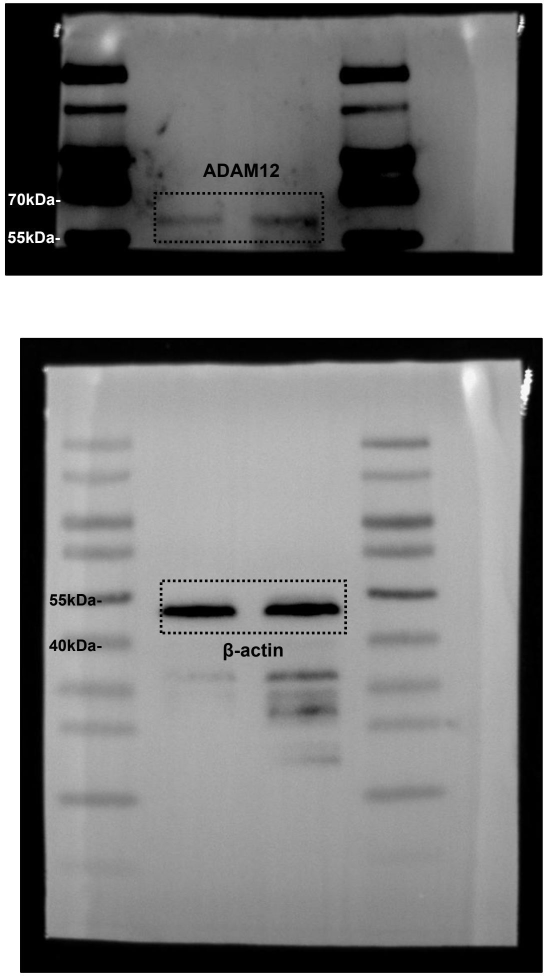

Figure S7C

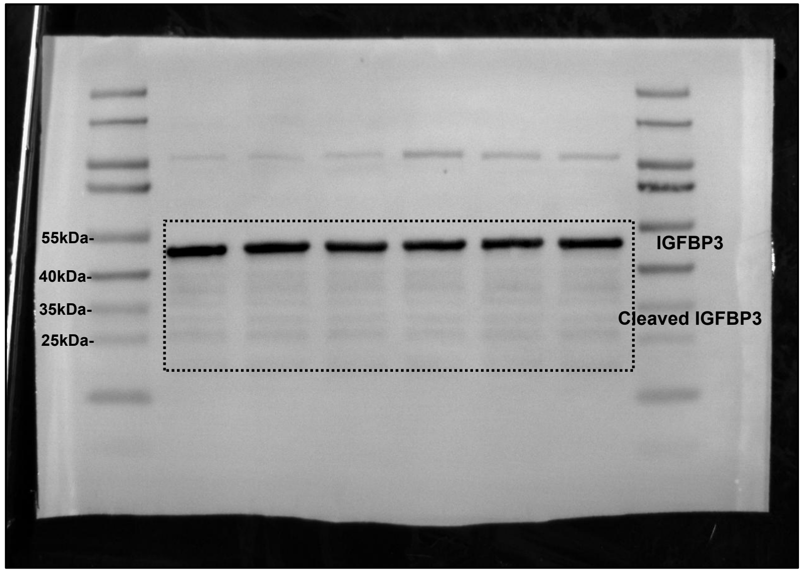

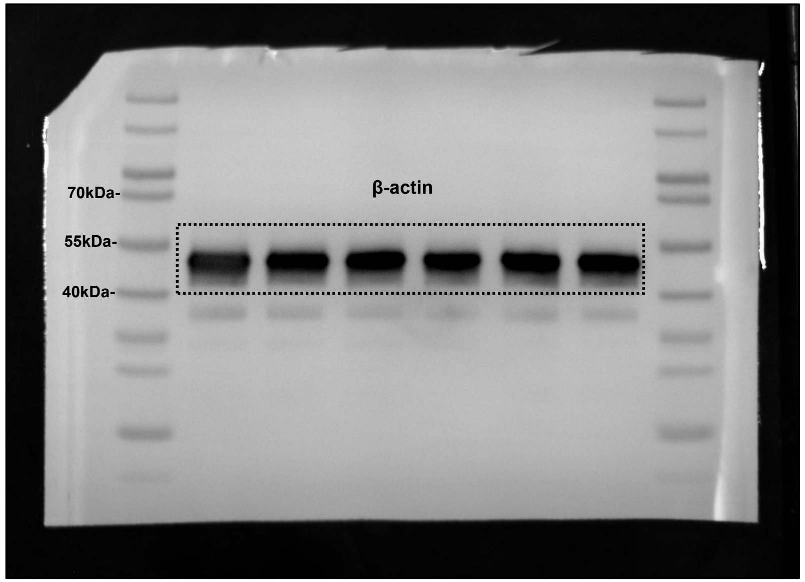

Figure S7D

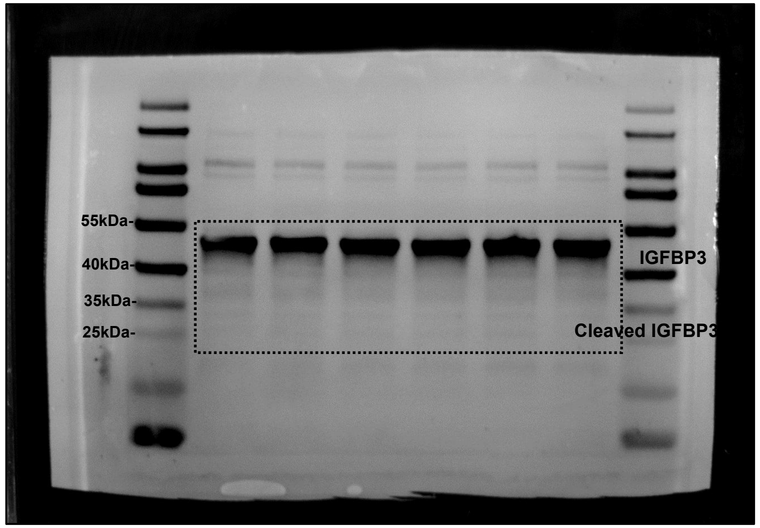

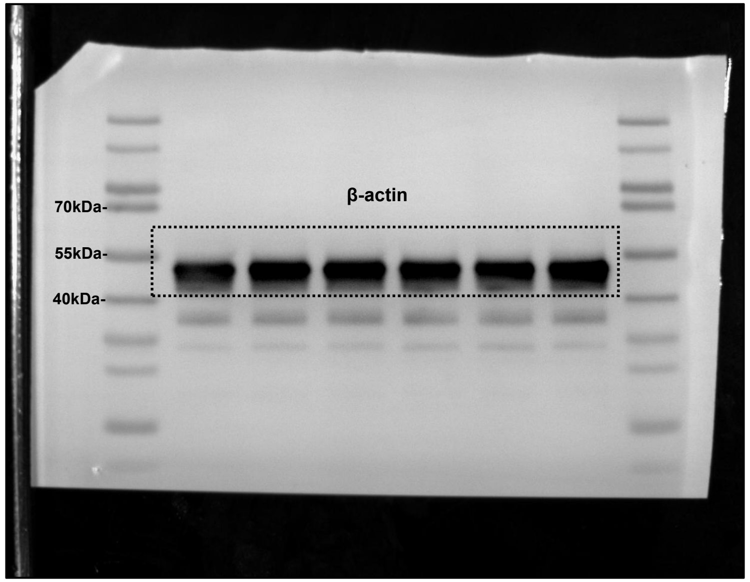

Figure S7G

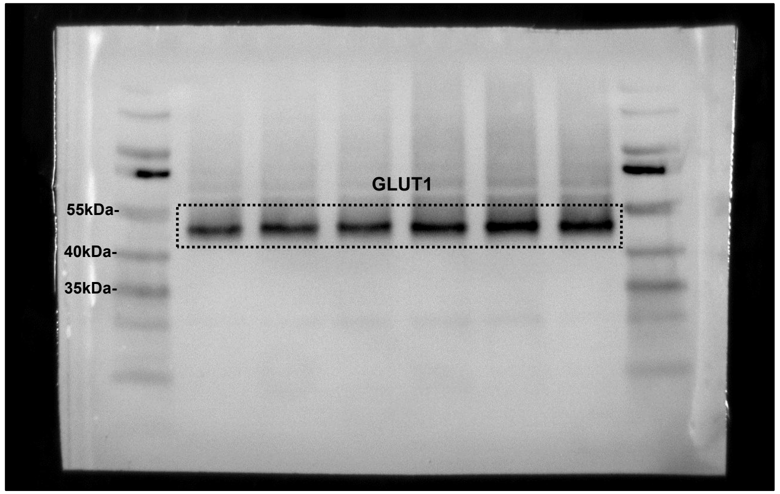
GLUT1
55kDa-
40kDa-
35kDa-

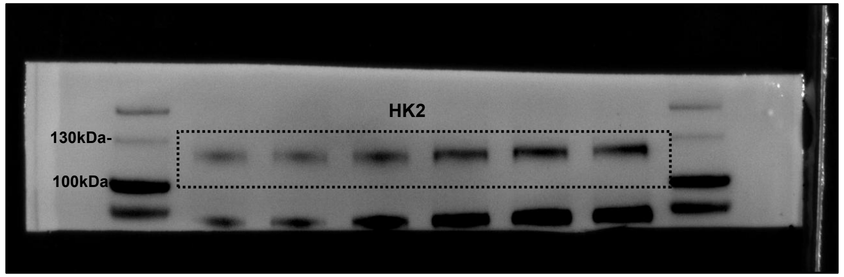
HK2
130kDa-
100kDa

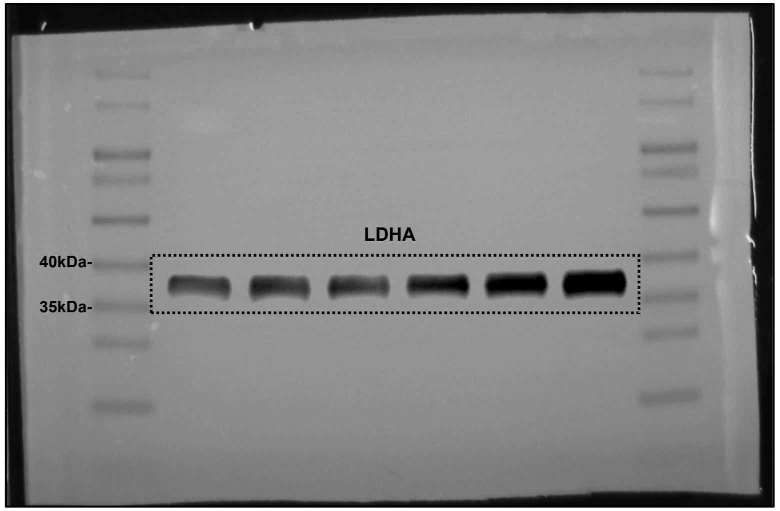
LDHA
40kDa-
35kDa-

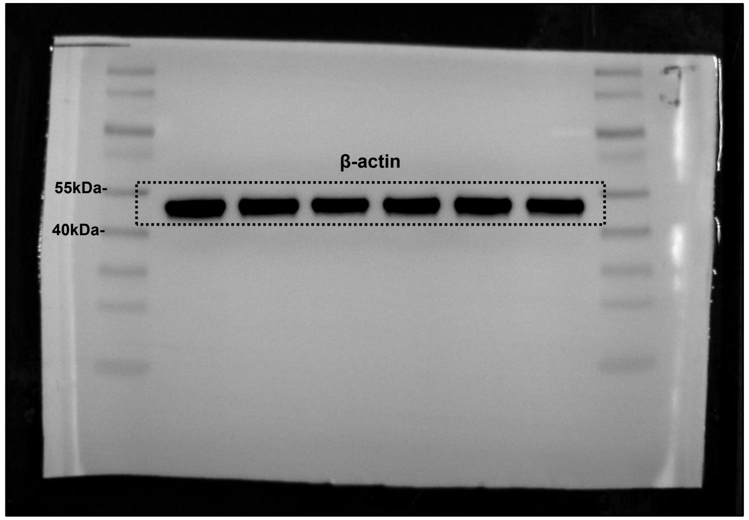
β-actin
55kDa-
40kDa-

Figure S8B

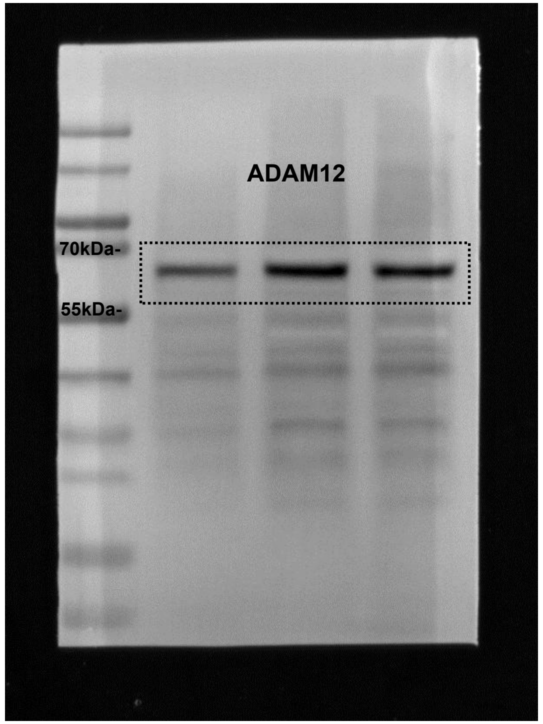

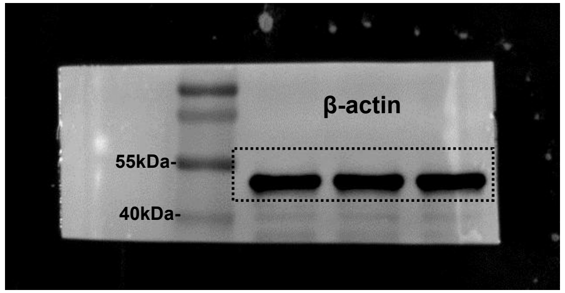

Figure S8G

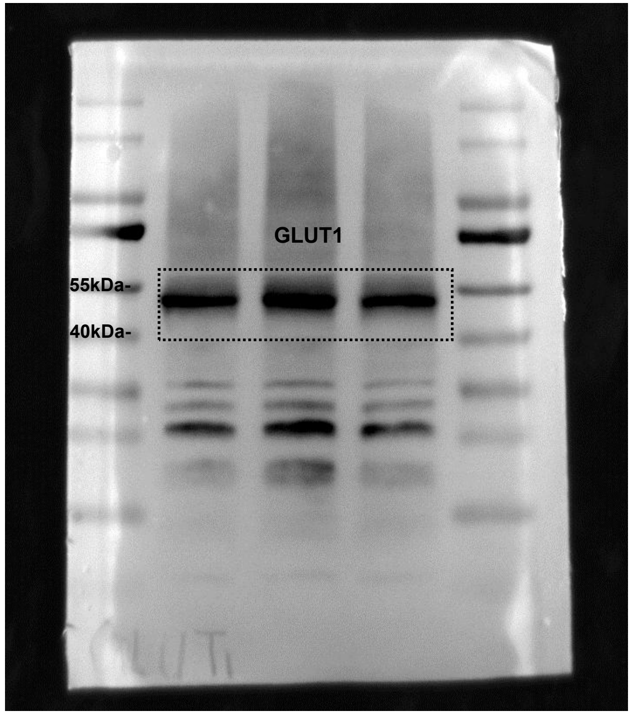

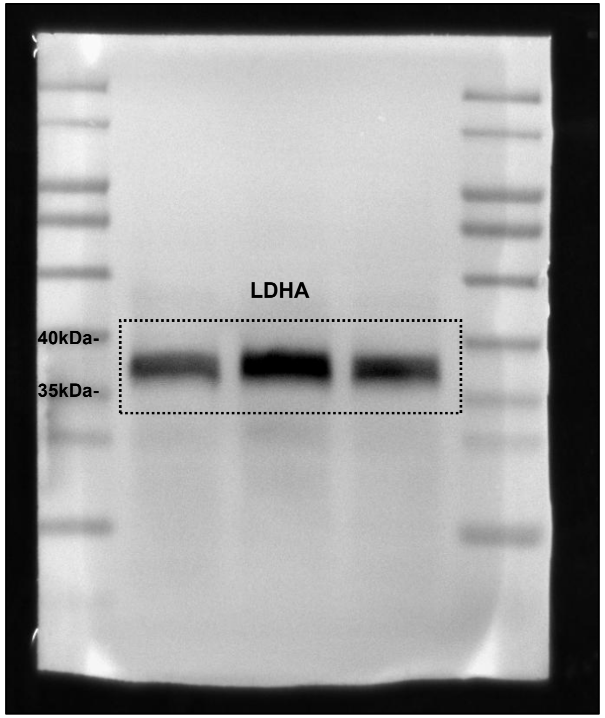

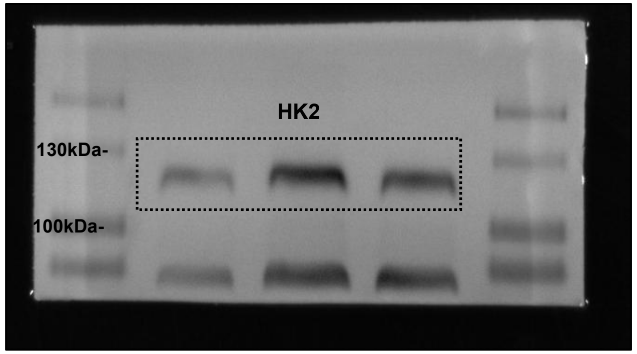

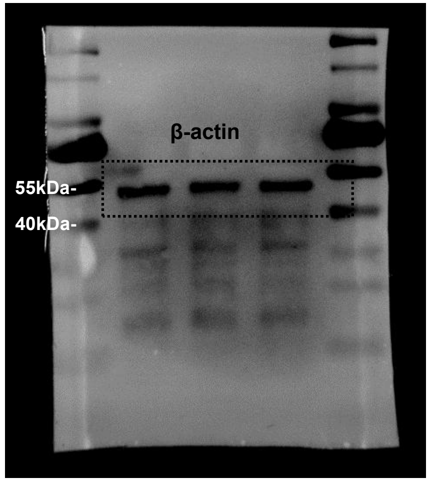

Supplement: Supplementary file 11 — Full and uncropped western blots [file 41420_2026_3044_MOESM11_ESM.pdf]
